# Diversity of the *Bosmina* (Cladocera: Bosminidae) in China, revealed by analysis of two genetic markers (mtDNA 16S and a nuclear ITS)

**DOI:** 10.1186/s12862-019-1474-4

**Published:** 2019-07-16

**Authors:** Liufu Wang, Hang Zhuang, Yingying Zhang, Wenzhi Wei

**Affiliations:** College of Animal Science and Technology, Yangzhou University, Yangzhou, 225009 China

**Keywords:** *Bosmina*, Distribution, Genetic investigation, China

## Abstract

**Background:**

China is an important biogeographical zone in which the genetic legacies of the Tertiary and Quaternary periods are abundant, and the contemporary geography environment plays an important role in species distribution. Therefore, many biogeographical studies have focused on the organisms of the region, especially zooplankton, which is essential in the formation of biogeographical principles. Moreover, the generality of endemism also reinforces the need for detailed regional studies of zooplankton. *Bosmina*, a group of cosmopolitan zooplankton, is difficult to identify by morphology, and no genetic data are available to date to assess this species complex in China. In this study, 48 waterbodies were sampled covering a large geographical and ecological range in China, the goal of this research is to explore the species distribution of *Bosmina* across China and to reveal the genetic information of this species complex, based on two genetic markers (a mtDNA 16S and a nuclear ITS). The diversity of taxa in the *Bosmina* across China was investigated using molecular tools for the first time.

**Results:**

Two main species were detected in 35 waterbodies: an endemic east Asia *B. fatalis*, and the *B. longirostris* that has a Holarctic distribution. *B. fatalis* had lower genetic polymorphism and population differentiation than *B. longirostris. B. fatalis* was preponderant in central and eastern China, whereas *B. longirostris* was dominated in western China. The third lineage (*B. hagmanni*) was only detected in a reservoir (CJR) of eastern China (Guangdong province). *Bosmina* had limited distribution on the Tibetan plateau.

**Conclusions:**

This study revealed that the biogeography of *Bosmina* appear to be affected by historical events (Pleistocene glaciations) and contemporary environment (such as altitude, eutrophication and isolated habitat).

**Electronic supplementary material:**

The online version of this article (10.1186/s12862-019-1474-4) contains supplementary material, which is available to authorized users.

## Background

China is an important biogeographical zone, where the genetic legacies of the Tertiary and Quaternary periods are abundant. On the one hand, the region (~ 25–50°N) in the warm temperate-subtropical zone retained moderately high temperatures and provided a good shelter environment for endemics during Tertiary climatic changes [1, 2]. On the other hand, the existence of different geographic refugia of Pleistocene (an era in the Quaternary period) did shelter varied lineages with diversity that are also affected by vicariance processes and allopatric speciation [3, 4]. At contemporary time, China, a vast territory with different North-South monsoons and East-West altitudes, has a complex geographic environment, the effect of which on the species distribution is also important. Therefore, many biogeographical studies have focused on the organisms of the region [5–8]. In particular, zooplankton, a group of freshwater invertebrate with a key role in the formation of biogeographical principles, attracted researchers’ attention [9–11]. Previous genetic analyses have supported the generality of endemism and provincialism, and reinforced the need for detailed regional studies of zooplankton [12, 13].

Species of the genus *Bosmina* Baird, 1845 (Anomopoda: Bosminidae) is small cladocerans (approximation 0.254~0.319 mm) that exist in lakes and reservoirs in every biogeographical region [14, 15]. The genus not only purifies the water by the highly efficient filtration of bacteria, organic detritus and phytoplankton (top-down control) in the waterbodies, but also is rich in protein and fat, which can be used as bait for fish (bottom-up control). Therefore, *Bosmina* plays an important role in energy flow, the carbon cycle and the nutrient salt mineralization of waters [16]. Moreover, *Bosmina* may achieve high biomass under a variety of conditions that are unfavorable for other cladocerans. And its dominant position has become increasingly more apparent in recent decades [17]. *Bosmina* is also among the best preserved zooplankton in limnological sediments due to their robust carapace, making them ideal paleolimnological indicators of ecosystem change [18, 19].

Three species of this genus were reported in China based on a morphological study, including *B. longirostris* O. F. Müller, 1785, *B. coregoni* Baird, 1857 and *B. fatalis* Burckhardt, 1924 [20]. Due to the phenotypic plasticity induced by biotic/abiotic signals and the intermediate hybrid morphology that is affected by introgression, assignment of taxa within the *Bosmina* based on the general phenotype is unreliable [21–23]. Fortunately, DNA sequencing of a standard gene region or “DNA barcoding” might speed a solution [24]. Recent studies based on DNA markers have contributed significantly in the resolution of taxonomic units within *Bosmina* and have allowed questions regarding the diversity and distribution of species to be readdressed in other Holarctic regions (mainly in Europe and in North America) [25, 26]. However, no genetic data are available to assess the biogeography of *Bosmina* in China, which is rich in endemics that are awaiting description [2].

In the present study, zooplankton samples were analyzed from 48 Chinese waterbodies (from 2 to 5135 m above sea level; from oligotrophic to eutrophic conditions), in which 35 populations of *Bosmina* was detected. Because the mtDNA gene (partial 16S rDNA, further abbreviated as 16S) is highly informative in biogeographic studies, and the nuclear gene region (internal transcribed spacer) is most informative for *Bosmina* that involve comparisons above the species level [19]. Both fragments were chosen as molecular markers to detect distribution of different lineages for *Bosmina* and to reveal the DNA molecular information of those lineages found in China.

## Methods

### Sampling methodology

Previous study showed that density of *Bosmina* is relatively high in summer and positively correlated with water temperature [27], so all samples were collected in summer (June, July and August) of 2016 across China. We sampled waterbodies to cover as much as possible in five major regions, which could fully represent different geographic and limnological types of waterbodies in China. Eastern Plain Region (EPR) is located in the middle and lower reaches of the Yangtze River, where most waterbodies have a high degree of eutrophication. Mongolia–Xinjiang Plateau (MXP) is located in arid and semiarid regions with a sandy/dusty weather and less precipitation. Northeast China region (NCR) is located in a humid and sub-humid area with a continental monsoon climate. Waterbodies in this region are rich in organic materials and humus in sediments. Yunnan–Guizhou Plateau (YGP) is located in the southwest of China, in a large karst area, and there are > 30 waterbodies with a low degree of eutrophication. Tibetan Plateau (QTP) is located at the highest altitude and is the most recently formed region, which is the least polluted area [28]. The sampling situation is as follows: nineteen of these waterbodies were located on the EPR, seven waterbodies were situated on the MXP, four waterbodies lied on the NCR, eight waterbodies were seated on the YGP, and ten remaining waterbodies were distributed on QTP (Table 1).

**Table 1:** Environmental variables of the studied waterbodies

Waterbody name (abbreviation)	Latitude (N)	Longitude (E)	Altitude (m)	WT (°C)	SD (m)	TP (μg L^−1^)	Chl. a (mg L^− 1^)	T.S.I.	Trophic level
Eastern Plain Region (EPR)									
Chao Lake (CAH)	31.43	117.22	10	26.6 ± 1.42	0.75 ± 1.17	80.15 ± 2.59	4.76 ± 0.59	67.77	Eutro
Changjiang Reservoir (CJR)	22.29	113.26	24	26.4 ± 1.48	0.68 ± 0.57	54.15 ± 0.60	4.46 ± 0.23	55.90	Eutro
Dong Lake (DOH)	30.33	114.24	39	24.9 ± 3.69	0.63 ± 1.63	72.77 ± 0.17	4.79 ± 0.73	61.26	Eutro
Dongping Lake (DPH)	35.59	116.12	41	18 ± 3.34	0.62 ± 3.89	69.10 ± 0.88	4.64 ± 1.76	59.18	Eutro
Dongting Lake (DTH)	29.22	113.05	27	18.7 ± 5.49	0.7 ± 1.70	82.03 ± 0.97	3.07 ± 0.24	61.23	Eutro
Gaoyou Lake (GYH)	32.52	119.24	5	26.8 ± 1.65	0.77 ± 0.95	66.63 ± 2.72	3.68 ± 0.54	60.27	Eutro
Hongze Lake (HZH)	33.26	118.15	10	25.5 ± 4.09	0.76 ± 1.70	75.85 ± 2.23	3.80 ± 3.22	63.45	Eutro
Luhun Reservoir (LHR)	34.22	112.01	540	23.6 ± 3.72	0.32 ± 2.40	50.00 ± 0.67	3.45 ± 3.04	38.89	Oligo
Luo Lake (LUH)	32.33	109.12	17	19.6 ± 4.50	0.63 ± 0.25	59.07 ± 85.08	1.02 ± 19.21	44.43	Oligo/Meso
Luoma Lake (LMH)	34.00	118.15	17	28.6 ± 3.97	0.6 ± 0.60	71.15 ± 8.77	3.51 ± 5.11	55.56	Eutro
Poyang Lake (PYH)	29.26	116.00	17	25.5 ± 4.92	0.61 ± 1.66	60.86 ± 3.13	4.84 ± 0.31	57.04	Eutro
Qiandao Lake (QDH)	29.4	119.05	600	23.8 ± 4.15	0.4 ± 2.33	75.55 ± 1.90	4.85 ± 1.03	54.76	Eutro
Songtao Reservoir (STR)	19.24	109.33	165	23.9 ± 4.32	0.5 ± 1.06	73.81 ± 0.38	4.71 ± 0.78	57.09	Eutro
Tai Lake (TAH)	31.29	120.07	2	26.6 ± 4.09	0.76 ± 0.92	71.57 ± 0.25	3.47 ± 0.22	60.91	Eutro
Xiliang Lak (XLH)	29.55	114.03	16	21.5 ± 4.66	0.64 ± 0.85	74.57 ± 1.13	3.91 ± 0.61	59.47	Eutro
Xinfengjiang Reservoir (XFJR)	23.43	114.38	119	23.8 ± 4.78	0.38 ± 0.78	80.04 ± 3.12	4.37 ± 2.09	54.15	Eutro
Ying Lake (YIH)	32.63	108.79	515	23.3 ± 3.64	0.77 ± 0.21	62.22 ± 1.73	3.80 ± 0.75	59.22	Eutro
Mongolia–Xinjiang Plateau (MXP)									
Bositeng Lake (BSTH)	41.54	86.44	1048	23.6 ± 3.68	0.5 ± 0.14	47.37 ± 0.95	1.10 ± 0.35	36.24	Oligo
Hulun Lake (HLH)	49.19	117.38	539	22.1 ± 5.73	0.73 ± 1.10	78.83 ± 0.89	1.70 ± 1.84	56.38	Eutro
Hasuhai Lake (HSH)	40.71	111.01	988	21.3 ± 6.00	0.61 ± 0.49	73.69 ± 4.57	3.84 ± 0.16	57.84	Eutro
Kundulun Lake (KDLH)	40.78	109.78	1067	22.3 ± 5.86	0.61 ± 4.63	40.00 ± 4.07	1.07 ± 2.39	37.46	Oligo
Liujiaxia Reservoir (LJXR)	35.56	103.19	1731	16.6 ± 5.83	0.62 ± 3.04	56.24 ± 2.27	1.02 ± 0.67	43.00	Oligo
TianChi (TIC)	43.53	88.07	1928	22.9 ± 3.25	0.43 ± 0.71	46.63 ± 2.89	1.10 ± 0.86	33.56	Oligo
Wulungu Lake (WLGH)	47.00	87.2	478	22.0 ± 3.17	0.55 ± 1.13	50.00 ± 1.00	1.84 ± 0.57	41.40	Oligo
Northeast China Region (NCR)									
Chagan Lake (CGH)	45.12	124.25	126	13.4 ± 6.29	0.77 ± 0.39	73.46 ± 4.11	2.79 ± 0.38	59.76	Eutro
Erlongshan Reservoir (ELSR)	45.43	127.24	720	24.7 ± 6.93	0.59 ± 0.28	60.00 ± 0.90	4.77 ± 0.64	55.87	Eutro
Songhua Lake (SHH)	43.39	126.46	261	29.8 ± 2.57	0.46 ± 0.18	77.99 ± 7.06	4.22 ± 0.08	55.47	Eutro
Yunnan–Guizhou Plateau (YGP)									
Ahang Reservoir (AHR)	26.32	106.39	1027	21.2 ± 2.45	0.6 ± 0.42	54.59 ± 1.14	4.66 ± 0.76	54.04	Eutro
Changshou Lake (CSH)	29.55	107.14	329	14.4 ± 8.63	0.62 ± 0.25	76.15 ± 6.44	2.69 ± 1.92	55.13	Eutro
Dianchi (DIC)	24.57	102.4	1886	22.9 ± 9.50	0.76 ± 0.25	93.56 ± 17.61	4.37 ± 2.17	71.11	Eutro
Fuxian Lake (FXH)	24.53	102.83	1722	21.8 ± 1.34	0.33 ± 2.17	30.00 ± 1.59	3.51 ± 2.03	32.88	Oligo
Hongfeng Lake (HFH)	26.32	106.25	1240	28.2 ± 2.14	0.61 ± 1.95	75.56 ± 1.88	3.37 ± 0.84	56.77	Eutro
Heilongtan Reservoir (HLTR)	30.02	104.03	568	26.8 ± 5.18	0.54 ± 2.66	72.35 ± 1.89	3.57 ± 2.57	54.22	Eutro
Sancha Reservoir (SCR)	30.18	104.16	457	20.2 ± 3.55	0.64 ± 2.10	74.15 ± 1.90	3.43 ± 1.38	57.56	Eutro
Yecheng Lake (YCH)	30.54	103.33	1600	23.1 ± 4.72	0.67 ± 3.63	63.92 ± 1.06	1.05 ± 2.92	47.20	Meso

Main environmental variables associated with trophic status: transparency, total phosphorus and chlorophyll-a, with also the value of trophic state index (T.S.I.) and the trophic level found. Limits of T.S.I.: Oligotrophic < 44; Mesotrophic between 44 and 54; Eutrophic > 54

### Environmental variables

Several variables were measured in situ: the sampling position (Latitude, Longitude and Altitude) was recorded with GPS-S6 (Newsmy), Secchi disk visibility (SD) was determined with a Secchi disk M369488 (Beijing Zhongxiyuanda Technology Co., LTD), and water temperature (WT) was measured using FG4-FK (Mettler Toledo Co., Greifensee, Switzerland). Water samples were collected with a Van Dorn bottle for subsequent laboratory analyses of total phosphorus (TP) concentrations, and chlorophyll-a (Chl. a) according to the methods described in detail by Huang [29]. Using the values of SD, TP, and Chl a, a classic trophic state index for waterbodies is calculated to characterize the trophic level of the sampled sites [30].

### Zooplankton sampling

**Fig. 1:**
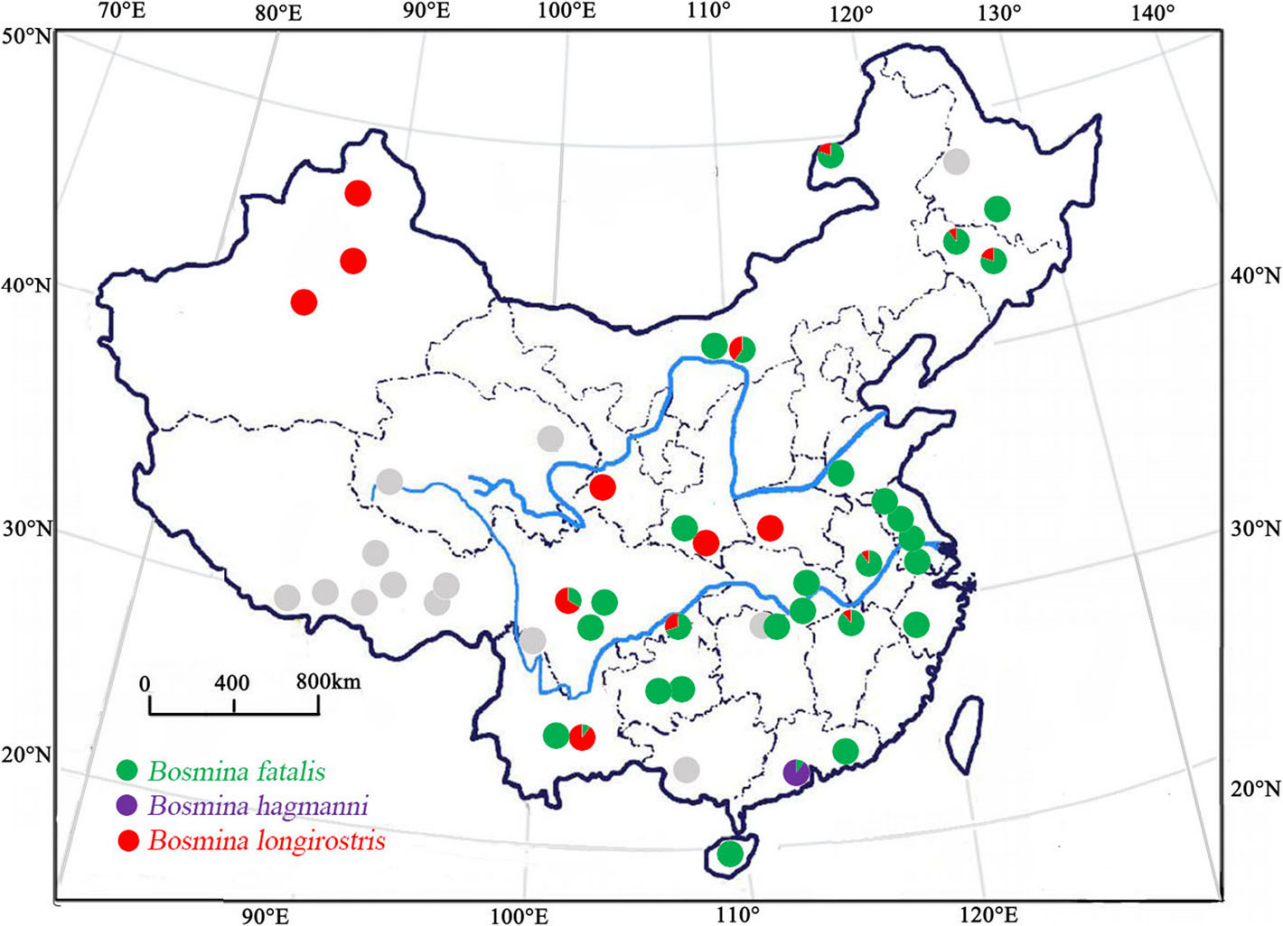
Geographic location of collection sites for *Bosmina*. Color dots: waterbodies inhabited with the *Bosmina*; gray dots: *Bosmina* was not detected. Species proportion in 35 sampled populations illustrated as a pie chart (Additional file 1: Table S1). Green symbols: *B. fatalis*; purple symbols: *B. hagmanni*; red symbols: *B. longirostris*. Taxon identification is based on 16S and ITS sequence variation

Zooplankton samples were collected (Additional file 1: Tables S1, S2 and Fig. 1) with a 125-mm plankton net hauled through the whole water column at three different sites per waterbody within the deep basin or from the shore; these samples were later pooled and preserved in 95% ethanol for further analyses. By using a stereomicroscope, *Bosmina* was found in 35 waterbodies and ten adult females were selected per waterbody for genetic analyses.

### Molecular analysis

DNA of the *Bosmina* specimens was extracted by the proteinase K method [31] and stored at − 4 °C. Each 50-μl polymerase chain reaction (PCR) consisted of 40 μl irradiated H_2_O, 5 μl 10× PCR buffer, 1.5 mM MgCl_2_, 2 mM of each dNTP, 1 μM of each primer, 0.5–1 units Taq DNA polymerase, and 1/10th DNA extract. The primers and PCR temperature profiles of the 16S and ITS followed those of a previous study [19]. Next, the PCR products were purified and sequenced in the forward direction on an ABI PRISM 3730 DNA capillary sequencer by Invitrogen Trading Co., Ltd. (China). The chromatograms were carefully checked for scoring errors.

### Sequence alignment

Sequences of the target individuals were aligned with reference species (Additional file 1: Tables S1 and S3) using the Clustal W algorithm [32] at locus 16S and ITS in MEGA.7 [33]. The genera of *Bosminopsis* and *Ilyocryptus* are distantly related to *Bosmina*; thus, the level of divergence between them could lead to an alignment error, long-branch attraction bias and incorrect model estimation. Therefore, in this study, model parameters and phylogenetic trees with in-group taxa only were estimated as suggested by Taylor et al. (2002) [19].

### Phylogeny

The phylogenetic relationships among lineages were visualized for both the 16S and ITS datasets. In cases where several individuals carried an identical haplotype, only one was included subsequently. First, the best-fit for both datasets (16S: GTR + G + I and ITS: GTR + G) were identified by jModelTest version 2.1.3 [34], and we then applied these models to run the maximum likelihood (ML) analysis in RAxML [35]. The assignment of species identity (or genetic lineage) of studied individuals was based on clustering with reference sequences in the phylogenetic trees, which was supported by 1000 bootstrapping. All subsequent calculations were based on the taxonomic units that were thereby defined.

### Genetic polymorphism and differentiation

The followed analyses were based on data (Additional file 1: Table S1) to learn the genetic polymorphism of two main lineages (i.e. *B. fatalis* and *B. longirostris*) found in this study (Fig. 1). In DnaSP v5, haplotype diversity (*Hd*) and nucleotide diversity (*Pi*) were calculated for the 16S (249 individuals) and the ITS (305 individuals) genes of main lineages, to determine the level of sequence diversity within the *Bosmina* [36]. Because 16S is more highly informative for biogeographic studies within species than ITS [19], we calculated *p*-distances (in MEGA.7) of 16S among all the geographical populations for *B. fatalis* and *B. longirostris* respectively, plus standard error calculated on 1000 bootstrap replicates [33]. In addition, the pairwise genetic differentiation of 16S among geographical populations was estimated (in Arlequin v. 3.11) using the fixation index *Fst*, which included information on mitochondrial haplotype frequency and genetic distances. The significance was tested by 1000 permutations for each pairwise comparison [37].

### Haplotype network

To describe the intraspecific variation and relationships among populations from different regions, two haplotype networks (16S and ITS) were constructed based on previous and our studies (Additional file 1: Tables S1 and S3) by HapView with the input of maximum-likelihood trees [38].

## Results

### Environmental data

Among the 35 sampling sites where *Bosmina* found, most central and eastern waterbodies (15 of EPR, 2 of MXP, 3 of NCR and 6 of YGP) usually have very high trophic levels, while western waterbodies (2 of EPR, 5 of MXP and 2 of YGP) are mesotrophic or oligotrophic (Table 1).

### Phylogeny and distribution

**Fig. 2:**
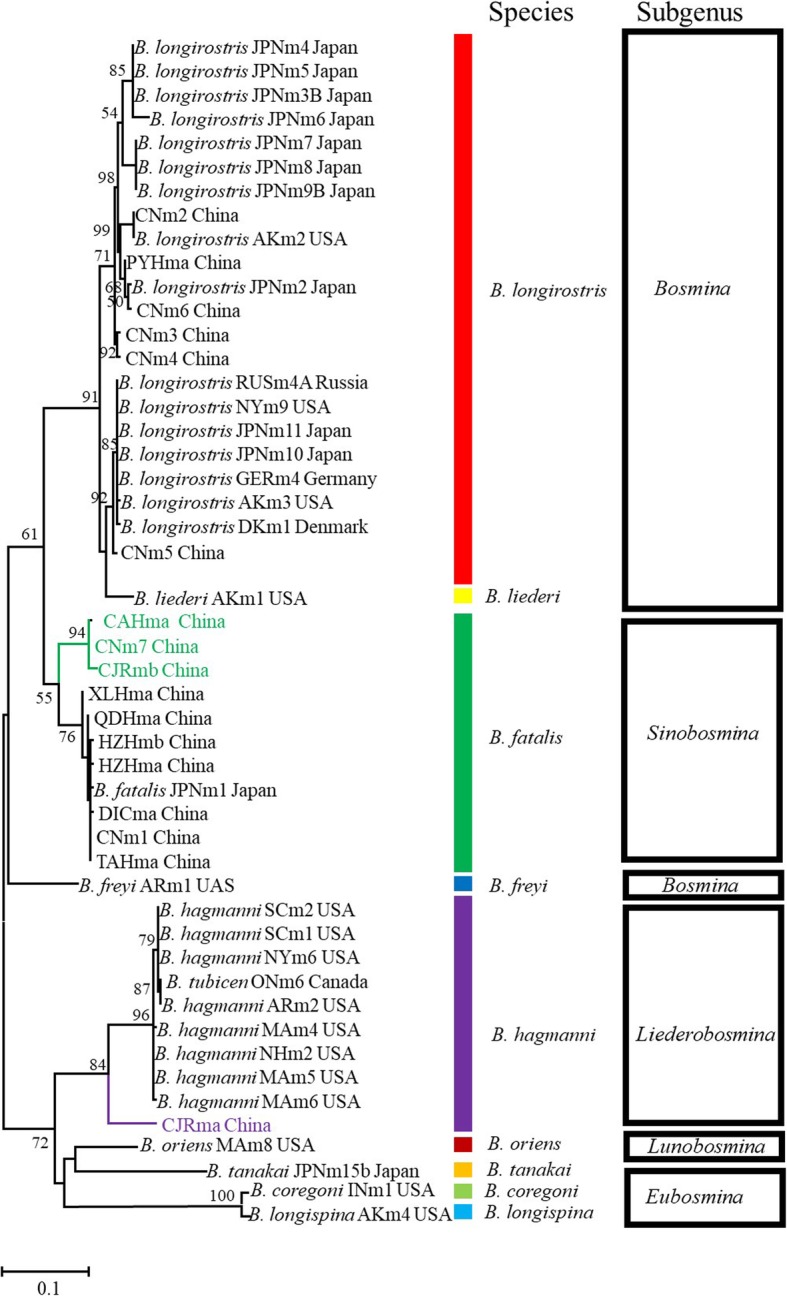
Maximum likelihood (ML) phylogenetic tree based on the 16S mitochondrial gene using RAxML. Codes of clones and a list of reference sequences are provided in the Additional file 1: Tables S1 and S3. More than 50% bootstrap support values are shown above the branches. The lineages (subclades) close to *B. fatalis* and *B. hagmanni* are marked in green and in purple, respectively. Colorful bands represent the classification of species, while black rectangles represent subgenera aggregation based on species

**Fig. 3:**
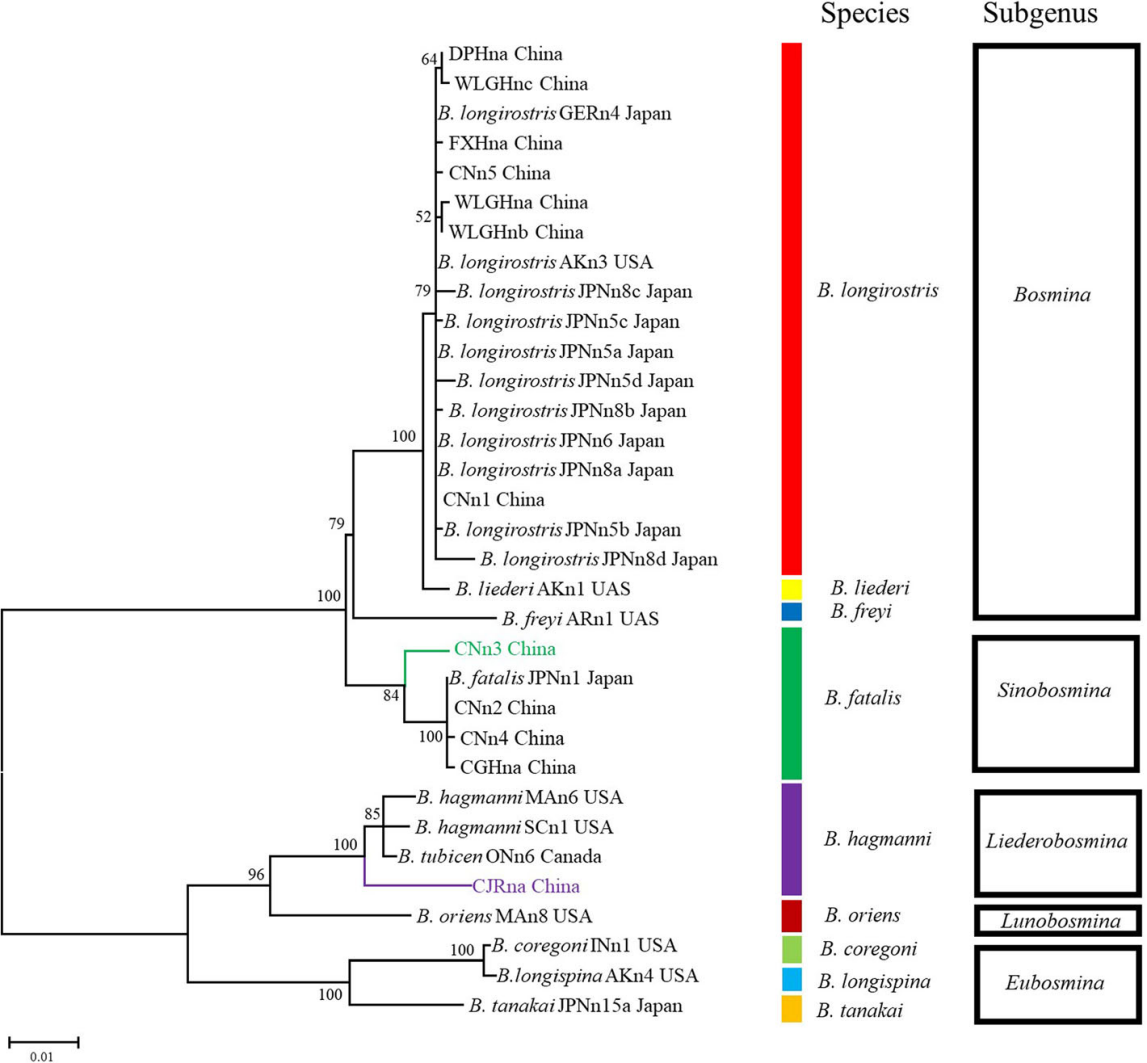
Maximum likelihood (ML) phylogenetic tree based on ITS nuclear gene using RAxML. Codes of clones and a list of reference sequences are provided in the Additional file 1: Tables S1 and S3. More than 50% bootstrap support values are shown above the branches. The lineages (subclades) close to *B. fatalis* and *B. hagmanni* are marked in green and in purple, respectively. Colorful bands represent the classification of species, while black rectangles represent subgenera aggregation based on species

Altogether, 249 individuals were successfully sequenced at the 16S locus, and 305 were sequenced at the ITS region (because of a failure in either amplification or sequencing; Table 1). Among these individuals, 17 unique 16S haplotypes and 12 unique ITS haplotypes were found (Additional file 1: Table S1). Phylogenetic analysis revealed that 291 out of 305 *Bosmina* individuals sampled in this study definitely belong to two species clades: *B. fatalis* and *B. longirostris.* Moreover, one subclade (with 5 individuals) was detected from *B. fatalis* and another (9 individuals) from the species of *B. hagmanni* (Figs. 2 and 3, Additional file 1: Table S1).

The *Bosmina* detected in China had different geographic distributions: *B. fatalis* was present in eutrophic waterbodies from central and eastern China, whereas *B. longirostris* was dominant in oligotrophic/mesotrophic waterbodies of the western region (Table 1 and Fig. 1). The lineage close to *B. fatalis* was distributed in four waterbodies, including CAH, CJR, DOH and PYH. The lineage close to *B. hagmanni* was rarely present in China from this study and was only detected in a reservoir (CJR) of the Guangdong province (Fig. 1 and Additional file 1: Table S1). No *Bosmina* was detected in 10 waterbodies on the Tibetan Plateau, indicating its limited distribution in the region (Fig. 1 and Additional file 1: Table S2).

### Genetic polymorphism and differentiation

The level of nucleotide polymorphism within *B. fatalis* was lower than *B. longirostris* at the 16S. However, this was not the case for the ITS, for which the level of nucleotide polymorphism between the two species was comparable (Table 2). The pairwise *p*-distance showed that most population of *B. fatalis* had lower differentiation than *B. longirostris*. In addition, most of the *Fst* population pairwise comparisons of *B. fatalis* were low and not significant. However, values of *Fst* in *B. longirostris* were generally high and most significant *Fst* values (*p* < 0.05) were included. The different *Fst* also displayed the different differentiation within *B. fatalis* and *B. longirostris* (Tables 3 and 4).

**Table 2:** Genetic diversity within Chinese samples from the *Bosmina*

	16S				ITS			
	*N*	*n*	*Hd*	*Pi*	*N*	*n*	*Hd*	*Pi*
*B. fatalis*	190	10	0.207	0.0092	210	4	0.078	0.00555
*B. longirostris*	59	6	0.789	0.0215	95	7	0.504	0.00590

Number individual (*N*), number of haplotypes (*n*), haplotype diversity (*Hd*) and nucleotide diversity (*Pi*)

**Table 3:**
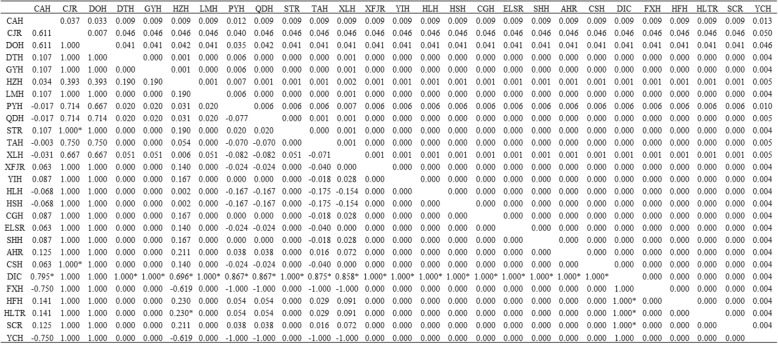
Genetic distance (*p*-distance: upper diagonal) and the fixation index considering genetic distances (*Fst*: lower diagonal) among 27 geographical populations of *B. fatalis* in China

*p < 0.05

**Table 4:** Genetic distance (*p*-distance: upper diagonal) and the fixation index considering genetic distances (*Fst*: lower diagonal) among 13 geographical populations of *B. longirostris* in China

	DPH	LHR	LUH	LJXR	PYH	BSTH	HSH	KDLH	TIC	WLGH	ELSR	CSH	FXH
DPH		0.003	0.002	0.006	0.017	0.034	0.002	0.012	0.034	0.039	0.017	0.017	0.002
LHR	0.415		0.001	0.008	0.017	0.033	0.004	0.010	0.033	0.039	0.017	0.017	0.004
LUH	0.143	−0.043		0.007	0.017	0.034	0.003	0.011	0.034	0.039	0.017	0.017	0.003
LJXR	0.059	0.664*	0.451*		0.015	0.034	0.005	0.014	0.034	0.039	0.015	0.015	0.005
PYH	0.464	1.000	0.571	0.536		0.034	0.017	0.016	0.034	0.041	0.004	0.004	0.017
BSTH	0.493*	0.666*	0.549*	0.513*	0.524		0.035	0.032	0.018	0.012	0.032	0.032	0.035
HSH	−0.013	1.000	0.543	−0.246	1.000	0.620*		0.014	0.035	0.039	0.017	0.017	0.000
KDLH	0.328*	0.250	0.208*	0.453*	0.444	0.393*	0.545*		0.032	0.041	0.018	0.018	0.014
TIC	0.493*	0.666*	0.549*	0.513*	0.524	−0.167	0.620	0.393*		0.012	0.032	0.032	0.035
WLGH	0.732*	1.000*	0.786*	0.768*	1.000	0.192	1.000*	0.696*	0.192		0.037	0.037	0.039
ELSR	0.569*	1.000*	0.650	0.573	1.000	0.620*	1.000	0.545*	0.620	1.000		0.000	0.017
CSH	0.569*	1.000	0.650*	0.573*	1.000	0.620*	1.000	0.545*	0.620	1.000*	0.000		0.017
FXH	−0.013	1.000	0.543	−0.246	1.000	0.620*	0.000	0.545*	0.620	1.000*	1.000	1.000	

*p < 0.05

### Haplotype network

**Fig. 4:**
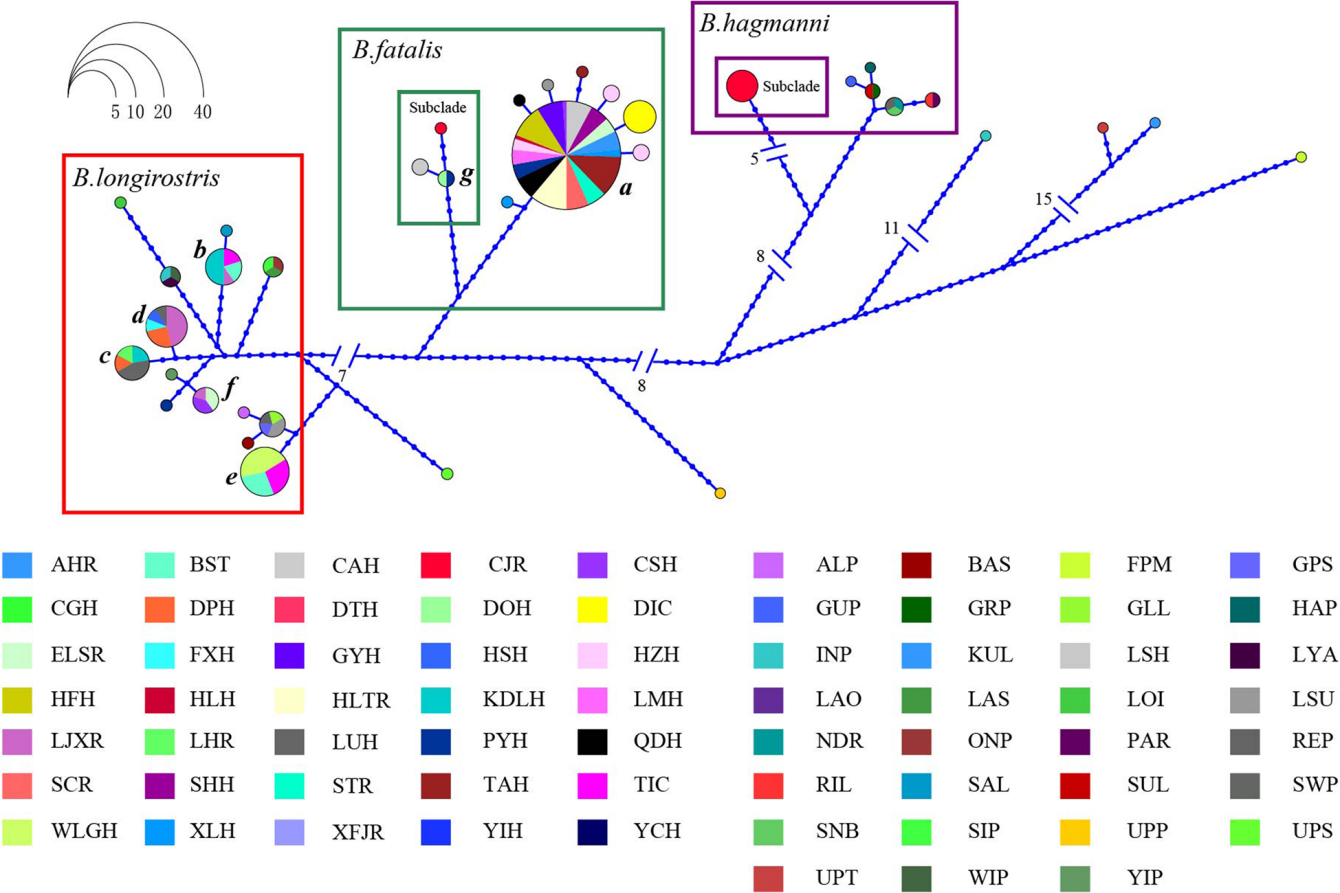
Haplotype networks of the 16S reconstructed in HapView. Circles represent haplotypes, with the sector size being proportional to the number of individuals; the lengths of the connecting lines reflect the number of mutations between them. The multicolored circles with letters *a-g* stand for shared haplotypes CNm1–7; the monochromatic circles stand for private haplotypes, whose subordinate waterbodies are shown in the legend (Table 1, Additional file 1: Tables S1 and S3)

**Fig. 5:**
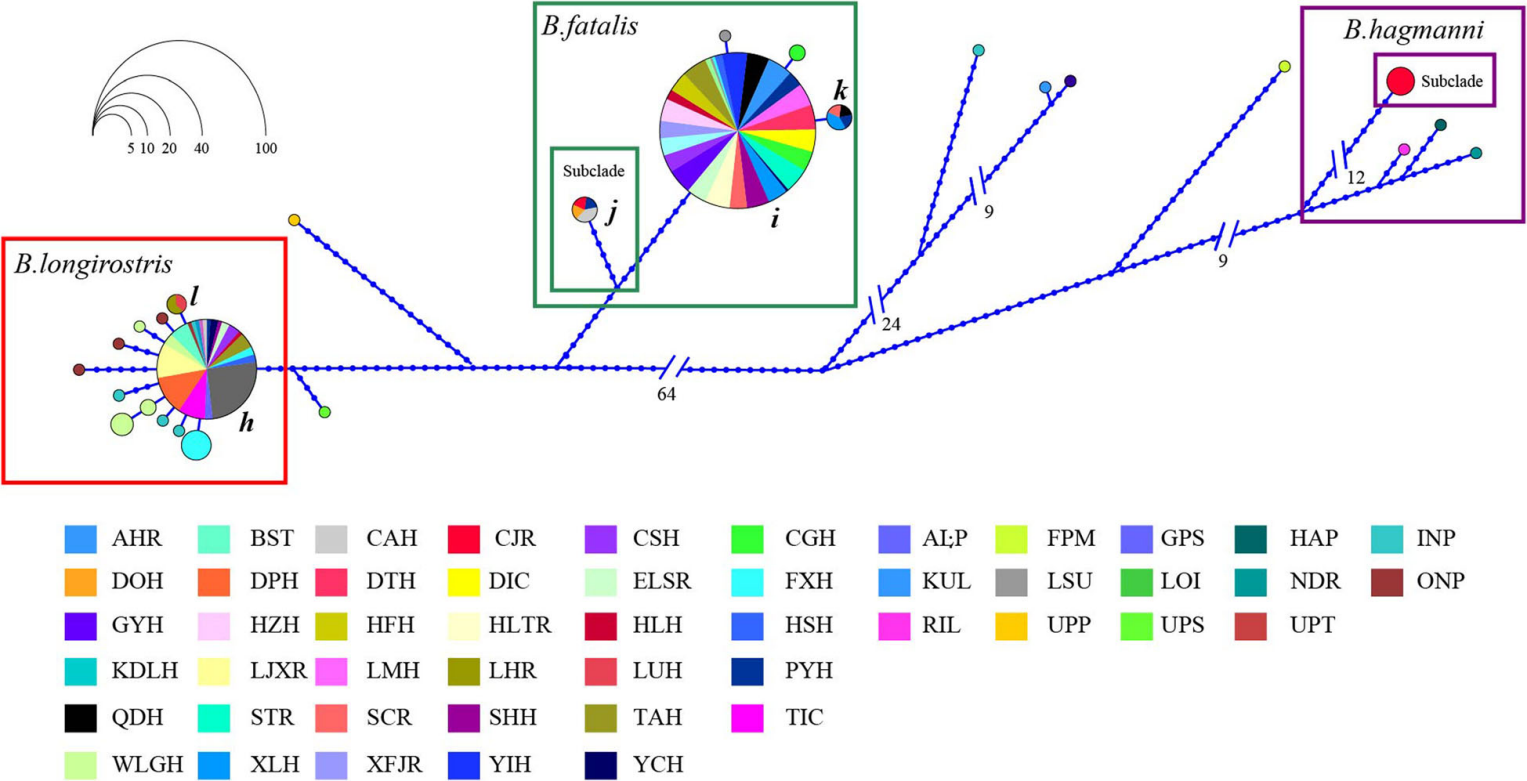
Haplotype networks of the ITS reconstructed in HapView using an ML tree. Circles represent haplotypes, with the sector size being proportional to the number of individuals; the lengths of the connecting lines reflect the number of mutations between them. The multicolored circles with letters *h-l* stand for shared haplotypes CNn1 to CNn6; the monochromatic circles stand for private haplotypes, whose subordinate waterbodies are shown in the legend (Table 1, Additional file 1: Tables S1 and S3)

The haplotypes of *B. fatalis* were restricted to eastern Asia (central and eastern China; Japan). In contrast, those of *B. longirostris* were widely distributed in other Holarctic regions (China, Denmark, Germany, Russia, Japan and the USA) (Figs. 4 and 5).

The 16S haplotype network of *B. fatalis* was arranged in a star-like pattern with a dominating haplotype (i.e., *a*) that was shared by 24 waterbodies, including CAH, DTH, GYH, HZH, LMH, PYH, QDH, STR, TAH, XLH, XFJR, YIH, HLH, HSH, CGH, ELSR, SHH, AHR, CSH, FXH, HFH, HLTR, SCR and YCH (waterbodies abbreviation were shown in Table 1). The 16S haplotype network of *B. longirostris* showed five shared haplotypes (i.e., *b*, *c*, *d*, *e*, *f*). CNm5 (*e*) was the most quantitatively frequent haplotype of *B. longirostris* and was shared by three waterbodies (BSTH, TIC and WLGH). The second-most frequent haplotypes were CNm3 (c) and CNm4 (d), which were found in four waterbodies (DPH, LHR, LUH and KDLH) and in five waterbodies (DPH, LUH, HSH, LJXR, and FXH), respectively (Fig. 4 and Additional file 1: Table S1).

The ITS haplotype network showed that *Bosmina* had the two (*i* and *h*) most abundant haplotypes. *B. fatalis* (*i*) was shared by 26 waterbodies (CAH, DOH, DTH, GYH, HZH, LMH, PYH, QDH, STR, TAH, XLH, XFJR, YIH, HLH, HSH, CGH, ELSR, SHH, AHR, CSH, DIC, FXH, HFH, HLTR, SCR, and YCH) and *B. longirostris* (*h*) was found in 17 waterbodies (CAH, DPH, LHR, LUH, PYH, BSTH, HLH, HSH, KDLH, LJXR, TIC, WLGH, ELSR, SHH, CSH, FXH, and YCH). In particular, *B. longirostris* (*h*), presented a frequently shared ITS haplotype with several haplotypes surrounded, showed the lower variation compared with 16S network (Fig. 5 and Additional file 1: Table S1).

## Discussion

### Different spatial pattern of *B. fatalis* and *B. longirostris*

Our phylogenetic analysis and network showed that the Chinese *B. fatalis* were closely related to those in Japan, confirming *B. fatalis* (also known as *Sinobosmina fatalis*) is endemic in Eastern Asia [39]. It has been early recognized that endemic taxa are not randomly distributed. Range restriction as a consequence of survival in refugia has been regarded as a driving force generating distributional patterns of endemics [12]. East Asia has been recognized as a major glacial Pleistocene refugium [40], where several new endemic cladocerans have found in Japan and neighboring areas [23, 41]. It seems that the endemism of *B. fatalis* is also a result of survival in refugia. The waves of Pleistocene glaciation, resulted in mass extinction of biotas in many region [1], while East Asia region remained comparatively unchanged [40]. Therefore, *B. fatalis* may survive in this region until now. In addition, the vicariance effects of the Pleistocene glaciations have played a major role in shaping the intraspecific divergence of freshwater zooplankton [12]. A lineage close to *B. fatalis* found in only four waterbodies (CAH, CJR, DOH and PYH) may be a geographically isolated species differentiated from *B. fatalis*.

Some cladocerans, such as *B. fatalis*, may be relicts with restricted-range (endemic), whereas others, commonly and widely distributed within their extensive primary ranges, possiblely are not [2]. We found close relatives among *B. longirostris* lineages from China, Denmark, Germany, Russia, Japan and the USA, which suggested that *B. longirostris* is widespread in the Holarctic region [42]. Therefore, *B. longirostris* should be an advanced lineage and have a non-relict status, which affected by current climate [43]. In all, the different spatial patterns of *B. fatalis and B. longirostris* can be explained by their different evolutionary status (relict and non-relict).

### Genetic polymorphism of *B. fatalis* and *B. longirostris*

Due to small population size and past bottlenecks, many relicts tend to have low within-population genetic polymorphism [44, 45]. Accordingly, the lower nucleotide diversity of *B. fatalis* than *B. longirostris* at 16S locus could be another evidence of their different evolutionary status (Table 2). Also, homogenization of refugial population after glaciation is unlikely in water fleas because they should possess pronounced refugial inertia. That is, successful colonization of an occupied water body is theoretically very difficult because of the strong priority effects from rapid lake-specific selection, massive egg banks and large existing populations [26]. The effect can essentially extend the time of genetic isolation of refugia even in the face of dispersal [46]. This may be why their difference of nucleotide diversity at 16S locus have been preserved to the present. Furthermore, this refugial inertia could provide more time for reproductive isolation [26]. Therefore, after thousands of years of unglaciated conditions, *B. fatalis* seems remain distinct and did not hybridize with *B. longirostris*, although they co-occur in some waterbodies (Fig. 1).

However, as a non-coding sequence which do not involve in the formation of the ribosome, ITS have small selective pressure and high divergent rate. For example, ITS evolution rate is 2.5 times of mitochondrial gene (COI) in *Tetranychus* [47] and 2 times of rDNA in *Nasonia* [48]. In this study, the nucleotide diversity for *B. fatalis* is similar to this index for *B. longirostris* at the ITS locus (Table 2). That may be an evolutionary result of ITS between *B. fatalis* and *B. longirostris*.

### Genetic differentiation of *B. fatalis* and *B. longirostris*

“Three Gradient Terrains” is a general description of Chinese terrain characters on relief amplitude. It portrays an outline of terrain changes like ladders along West-East direction. The first gradient terrain has the highest topography, with an altitude of over 2,600 m, mainly on the QTP. The second step is between 660 m and 2,600 m above sea level, with many large plateaus and basins. The third stair is below 660 m above sea level. In this study, *B. fatalis* was on the third stair (Table 1), where hills, low mountains and plains are interlaced [49]. The lower and similar altitude of waterbodies is good for gene communication between different population of zooplankton [9]. That may be the reason for the lower differentiation within *B. fatalis* than *B. longirostris* (Tables 3 and 4). On the other hand, it has reported that the geological isolation induced by different altitude can limit gene flow between subpopulation of zooplankton and lead to their differentiation [50]. *B. longirostris* was distributed on both the first and second steps (Table 1). The topographic drop between these steps, about 1000 m, reflects a strong gradient nature and may limit gene flow [49]. That may be responsible for the higher differentiation within *B. longirostris* (Tables 3 and 4). Due to differing mutation rates and increased rate of transitional saturation rate, 16S will be more highly informative in biogeographic differentiation than ITS. Therefore, the higher differentiation may be shown in 16S, but not in ITS [19]. That should be explained for the different patterns of the haplotype networks obtained for *B. longirostris* at 16S and ITS genes (Figs. 4 and 5).

### Different ecological preponderance of *B. fatalis* and *B. longirostris*

A number of physical, chemical and biological factors in the habitat can influence the community structure of zooplankton, and the factors recognized as the most important by majority of investigators were temperature, the quality and availability of food, competition and predation [50–52]. Nevertheless, there were no marked differences between the growths of *B. fatalis* and *B. longirostris* in response to temperatures ranging from 10° to 30 °C in a laboratory experiment [53]. Our temperature data of sampled waterbodies did not exceed this range (13.4 °C to 29.8 °C) (Table 1), which indicated that food, competition or predation seemed to more directly affect the population densities of *B. fatalis* and *B. longirostris*. The central and eastern waterbodies in our dataset (those on the NCR, YGP and the eastern of MXP and EPR) usually had very high trophic levels, while western waterbodies (those on the western of MXP and EPR) were mostly mesotrophic or oligotrophic and were less affected by human activities (Table 1). Eutrophication often causes an increased density of phytoplankton, especially the bloom of Microcystis [28]. In such condition, *B. fatalis* can overcome *B. longirostris* because of more efficient capture and utilization [54]. On the other hand, eutrophication favours cyclopoids, which can prey on *Bosmina* [55, 56]. Previous study has shown that *B. fatalis* is a superior competitor against *B. longirostris* and is more resistant to predation [57]. Therefore, competitive edge induced by food and predation in eutrophic waterbodies can be responsible for the preponderance of *B. fatalis* in the central and eastern region. On the contrary, dominance of *B. longirostris* was observed in western waterbodies, which are mesotrophic/oligotrophic.

### Appearance of *B. hagmanni* in China

We only found one lineage close to *B. hagmanni* in a single locality, the Changjiang Reservoir (CJR) in Guangdong province. *B. hagmanni* was thought to be confined to the American continent [58]. It seems that an American local lineage of *B. hagmanni* spread to the new continent, which is not surprising, since previous studies have shown that *Bosmina* can invade new continents by ship [59]. Guangdong province, which is in frequent maritime trade with America, may provide convenient conditions for alien *Bosmina* invasion [60]. Nevertheless, the invasion range in China should be quite limited (only present in one reservoir of our samples). A previous study suggested that the interaction between the trophic state and species identity influenced the invasion success of Cladocera [61, 62]. Although the waterbodies of Guangdong province are indeed eutrophic [63], we have little knowledge regarding the biological identity of *B. hagmanni*. Whether eutrophication also restricted the invasion range of *B. hagmanni* could be an interesting issue for future studies.

### The limited distribution of Bosmina on the Tibetan plateau

Although previous reports showed that *Bosmina* is a cosmopolitan genus of Cladocera, we did not find any species in 10 waterbodies of Tibetan Plateau, where the waterbodies have relatively limited phytoplankton for planktivorous zooplankton [28]. Due to the phenotypic plasticity of the filter screens, *Daphnia* adapted to the low-food environment [64] and may generate the effective monopoly of food resources, thereby avoiding the spread of other zooplankton [46]. The limited distribution of *Bosmina* on the Tibetan Plateau may be relative to the extensive distribution of *Daphnia* in the same region [11]. Moreover, the Tibetan Plateau, as the roof of the world (an elevation of more than 4,000 m, average above sea level) surrounded by high mountains, is ecologically isolated from other regions of China [65]. The more isolated the habitat is, the smaller the probability is that species will colonize it, especially such species as zooplankton that disperse by passive means [66], which may be another reason why *Bosmina* spread into the Tibetan Plateau finitely.

## Conclusion

Based on two genetic markers (mtDNA 16S and a nuclear ITS), we identified individuals from the *Bosmina* that were sampled from 35 Chinese waterbodies as descendants of two primary species (*B. fatalis* and *B. longirostris*) with different spatial pattern, which may be related to their different evolutionary status (relict and non-relict). Their different genetic polymorphism also seems to an inertia of their own evolutionary status. Nevertheless, their different genetic differentiation may be related to the altitude of their habitat. Furthermore, *B. fatalis* was preponderant in central and eastern China, whereas *B. longirostris* was dominant in western China. Their different ecological dominance may result from competitive interaction induced by food and predation in different trophic waterbodies. Among the waterbodies sampled, the lineage close to alien *B. hagmanni* was found only at a single locality (CJR), which might be invaded. The limited distribution of *Bosmina* on the Tibetan Plateau is a feature, which could be explained by resource monopoly of *Daphnia* in isolated habitat.

## Additional files

Additional file 1: **Table S1.** Numbers of observed haplotypes (of the 16S and ITS) in the investigated Chinese waterbodies; **Table S2.** The waterbodies where *Bosmina* was not found; **Table S3.** List of GenBank reference clones from Japanese, North American, European, Southern and Central Asian specimens used in phylogenetic analyses and haplotype networks. (DOCX 27 kb)

Additional file 2: The obtained haplotypes of 16S and ITS in this study. (DOCX 16 kb)

## Data Availability

The datasets supporting the conclusions of this article are included in this article and its additional files. In particular, Additional file 2 (the sequence data used in this study) is submitting to the GenBank databases under accession numbers: MH918683-MH918699 for 16S and MH918700-MH918711 for ITS.

## Abbreviations

16S: Partial 16S rDNA
AHR: Ahang Reservoir
BSTH: Bositeng Lake
CAH: Chao Lake
CGH: Chagan Lake
Chl. a: Chlorophyll-a
CJR: Changjiang Reservoir
CSH: Changshou Lake
DIC: Dianchi
DOH: Dong Lake
DPH: Dongping Lake
DTH: Dongting Lake
ELSR: Erlongshan Reservoir
EPR: Eastern Plain Region
FXH: Fuxian Lake
GYH: Gaoyou Lake
HFH: Hongfeng Lake
HLH: Hulun Lake
HLTR: Heilongtan Reservoir
HSH: Hasuhai Lake
HZH: Hongze Lake
ITS: Internal transcribed spacer
KDLH: Kundulun Lake
LHR: Luhun Reservoir
LJXR: Liujiaxia Reservoir
LMH: Luoma Lake
LUH: Luo Lake
MXP: Mongolia–Xinjiang Plateau
NCR: Northeast China Region
PYH: Poyang Lake
QDH: Qiandao Lake
QTR: Tibetan Plateau
SCR: Sancha Reservoir
SD: Secchi disk visibility
SHH: Songhua Lake
STR: Songtao Reservoir
T.S.I.: Trophic state index
TAH: Tai Lake
TIC: TianChi
TP: Total phosphorus
WLGH: Wulungu Lake
WT: Water temperature
XFJR: Xinfengjiang Reservoir
XLH: Xiliang Lak
YCH: Yecheng Lake
YGP: Yunnan–Guizhou Plateau
YIH: Ying Lake

## References

[1] Sen LC, Fei WY, Gao SQ (2001). Climate analysis of endemic species—a novel method for quantitative analysis of global climate change since tertiary. Acta Bot Sin.

[2] Korovchinsky NM (2006). The Cladocera (Crustacea: Branchiopoda) as a relict group. Zool J Linnean Soc.

[3] Willis KJ, Whittaker RJ (2000). The refugial debate. Science..

[4] Ding L, Gan XN, He SP (2011). A phylogeographic, demographic and historical analysis of the short-tailed pit viper (*Gloydius brevicaudus*): evidence for early divergence and late expansion during the Pleistocene. Mol Ecol.

[5] Zhang HH, Dou HL, Chen L (2015). Genetic diversity and phylogenetic analysis of wolves from different geographical regions of China. Acta Ecol Sin.

[6] Dufresnes C, Litvinchuk SN, Borzee A (2016). Phylogeography reveals an ancient cryptic radiation in east-Asian tree frogs (*Hylajaponica group*) and complex relationships between continental and island lineages. BMC Evol Biol.

[7] Yang QW, Huang J (2013). Research progress on genetic diversity of *Oryza rufipogon* in China. Acta Agron Sin.

[8] Zhang YH, Wang IJ, Comes HP (2016). Contributions of historical and contemporary geographic and environmental factors to phylogeographic structure in a tertiary relict species, *Emmenopterys henryi* (Rubiaceae). Sci Rep.

[9] Ma X, Petrusek A, Wolinska J (2015). Diversity of the *Daphnia longispina* species complex in Chinese lakes: a DNA taxonomy approach. J Plankton Res.

[10] Taylor DJ, Finston TL, Hebrt PDN (1998). Biogeography of a widespread freshwater crustacean: pseudocongruence and cryptic endemism in the north american *Daphnia laevis* complex. Evolution..

[11] Xu L, Lin Q, Xu S (2018). *Daphnia* diversity on the Tibetan plateau measured by DNA taxonomy. Ecol Evol.

[12] Xu S, Hebert PDN, Kotov AA (2009). The noncosmopolitanism paradigm of freshwater zooplankton: insights from the global phylogeography of the predatory cladoceran *Polyphemus pediculus* (Linnaeus, 1761) (Crustacea, Onychopoda). Mol Ecol.

[13] Adamowicz SJ, Hebert PDN, Marinone MC (2004). Species diversity and endemism in the *Daphnia* of Argentina: a genetic investigation. Zool J Linn Soci.

[14] Goulden CE, Frey DG (1963). The occurrence and significance of lateral head pores in the genus *Bosmina* (Cladocera). Int Rev Hydrobiol.

[15] Havens K, Decosta J (1985). The effect of acidification in enclosures on the biomass and population size structure of *Bosmina longirostris*. Hydrobiologia..

[16] Huibin LU, Chen G, Chen X (2015). The long-term effects of bottom-up and top-down forcing on zooplankton: an example from sedimentary Bosminid records of lake dianchi and lake fuxian. J Lake Sci..

[17] Yun L, Ping X, Zhao D (2016). Eutrophication strengthens the response of zooplankton to temperature changes in a high-altitude lake. Ecol Evol..

[18] Kerfoot WC (1981). Long-term replacement cycles in cladoceran communities: a history of predation. Ecology..

[19] Taylor DJ, Ishikane CR, Haney RA (2002). The systematics of Holarctic bosminids and a revision that reconciles molecular and morphological evolution. Limnol Oceanogr.

[20] Chiang SC, Du NS (1979). Fauna sinica; crustacea: freshwater cladocera.

[21] Lieder U (1983). Introgression as a factor in the evolution of polytypical plankton Cladocera. Int Rev Hydrobiol.

[22] Sakamoto M, Hanazato T (2009). Proximate factors controlling the morphologic plasticity of *Bosmina*: linking artificial laboratory treatments and natural conditions. Hydrobiologia..

[23] Kotov AA, Ishida S, Taylor DJ (2009). Revision of the genus *Bosmina* Baird, 1845 (Cladocera: Bosminidae), based on evidence from male morphological characters and molecular phylogenies. Zool J Linnean Soc.

[24] Costa FO, Dewaard JR, Boutillier J (2007). Biological identifications through DNA barcodes: the case of the Crustacea. Can J Fish Aquat Sci.

[25] Faustova M, Sacherova V, Svensson J-E (2011). Radiation of European *Eubosmina* (Cladocera) from *Bosmina(E.) longispina*—concordance of multipopulation molecular data with paleolimnology. Limnol Oceanogr.

[26] Haney RA, Taylor DJ (2003). Testing paleolimnological predictions with molecular data: the origins of Holarctic *Eubosmina*. J Evol Biol.

[27] Adamczuk M, Mieczan T, Tarkowska-Kukuryk M (2015). Rotatoria–Cladocera–Copepoda relations in the long-term monitoring of water quality in lakes with trophic variation (E. Poland). Environ Earth Sci.

[28] Wang SM, Dou HS (1995). Record of China Lakes.

[29] Huang XF (1999). Survey, observation and analysis of lake ecology.

[30] Carlson RE (1977). A trophic state index for lakes. Limnol Oceanogr.

[31] Schwenk K, Sand A, Boersma M (1998). Genetic markers, genealogies and biogeographic patterns in the Cladocera. Aquat Ecol.

[32] Thompson JD, Higgins DG, Gibson TJ (1994). CLUSTALW: improving the sensitivity of progressive multiple sequence alignment through sequence weighting, position-specific gap penalties and weight matrix choice. Nucleic Acids Res.

[33] Kumar S, Stecher G, Tamura K (2016). Mega7: molecular evolutionary genetics analysis version 7.0 for bigger datasets. Mol Biol Evol.

[34] Darriba D, Taboada GL, Doallo R (2012). jModeltest 2: more models, new heuristics and parallel computing. Nat Methods.

[35] Stamatakis A, Hoover P, Rougemont J (2008). A rapid bootstrap algorithm for the RAxML web servers. Syst Biol.

[36] Librado P, Rozas J (2009). DnaSP v5: a software for comprehensive analysis of DNA polymorphism data. Bioinformatics..

[37] Excoffier L, Laval G, Schneider S (2005). Arlequin (version 3.0): An integrated software package for population genetics data analysis. Evol Bioinform.

[38] Salzburger W, Ewing GB, Haeseler AV (2011). The performance of phylogenetic algorithms in estimating haplotype genealogies with migration. Mol Ecol.

[39] Lieder U (1983). Revision of the genus *Bosmina* Baird, 1845 (Crustacea, Cladocera). Int Rev Hydrobiol.

[40] Krehenwinkel H, Graze M, Rodder D (2016). A phylogeographical survey of a highly dispersive spider reveals eastern Asia as a major glacial refugium for Palaearctic fauna. J Biogeogr.

[41] Ishida S, Kotov AA, Taylor DJ (2006). A new divergent lineage of *Daphnia* (Cladocera: Anomopoda) and its morphological and genetical differentiation from *Daphnia curvirostris* Eylmann. Zool J Linnean Soc.

[42] Adamczuk M (2016). Past, present, and future roles of small cladoceran *Bosmina longirostris* (O. F. müller, 1785) in aquatic ecosystems. Hydrobiologia.

[43] Nevalainen L, Luoto TP, Kultti S (2013). Spatio-temporal distribution of sedimentary Cladocera (Crustacea: Branchiopoda) in relation to climate. J Biogeogr.

[44] Chang C-S, Kim H, Park TY (2004). Low levels of genetic variation among southern peripheral poulations of the threatened herb, *Leontice microrhyncha* (Berberidaceae) in Korea. Biol Conserv.

[45] Hampe A, Jump AS (2011). Climate relicts: past, present, future. Annu Rev Ecol Evol Syst.

[46] De Meester L, Gomez A, Okamura B (2002). The monopolization hypothesis and the dispersal-gene flow paradox in aquatic organisms. Acta Oecol.

[47] Navajas M, Lagnel J, Gutierrez J (1998). Species wide homogeneity of nuclear ribosomal ITS2 sequences in the spider mite *Tetranychus urticae* contrasts with extensive mitochondrial COI polymorphism. Heredity..

[48] Campbell BC, Steffen-Campbell JD, Werren HJ (1993). Phylogeny of the *Nasonia* species complex (Hymenoptera: Pteromalidae) inferred from an internal transcribed spacer (ITS2) and 28S rDNA sequences. Insect Mol Biol.

[49] Jie J, Xin Y (2009). Quantitative segmentation of the three gradient terrain of China based on DEM. Geomatics World.

[50] SAMPAIO E. V., ROCHA O., MATSUMURA-TUNDISI T., TUNDISI J. G. (2002). Composition and abundance of zooplankton in the limnetic zone of seven reservoirs of the Paranapanema River, Brazil. Brazilian Journal of Biology.

[51] Johannsson OE, Robert OG (1991). Roles of predation, food, and temperature in structuring the epilimnetic zooplankton populations in Lake Ontario, 1981–1986. T Am Fish Soc.

[52] Nowicki Carly J., Bunnell David B., Armenio Patricia M., Warner David M., Vanderploeg Henry A., Cavaletto Joann F., Mayer Christine M., Adams Jean V. (2017). Biotic and abiotic factors influencing zooplankton vertical distribution in Lake Huron. Journal of Great Lakes Research.

[53] Hanazato T, Masayuki Y (1985). Effect of temperature in the laboratory studies on growth, egg development and first parturition of five species of Cladocera. Jpn J Limnol.

[54] Hanazato T, Yasuno M (1987). Experimental studies on competition between *Bosmina longirostris* and *Bosmina fatalis*. Hydrobiologia..

[55] Maier G (1998). Differential success of cyclopoid copepods in the pelagic zone of eutrophic lakes. J Marine Sys.

[56] Kerfoot WC (1978). Combat between predatory copepods and their prey: *Cyclops*, *Epischura*, and *Bosmina*. Limnol Oceanogr.

[57] Chang KH, Hanazato T (2004). Predation impact of *Leptodora kindtii* on population dynamics and morphology of *Bosmina fatalis* and *B. longirostris* in mesocosms. Freshw Biol.

[58] Beaver JR, Renicker TR, Tausz CE (2018). Distribution of six taxa in the family Bosminidae Baird (Crustacea: Branchiopoda: Anomopoda) in the plankton of lakes and reservoirs within the continental United States, including expanded range of the invasive cladoceran *Bosmina (Eubosmina) coregoni* Baird. Zootaxa..

[59] Santagata S, Gasiūnaite ZR, Verling E (2008). Effect of osmotic shock as a management strategy to reduce transfers of non-indigenous species among low-salinity ports by ships. Aquat Invasions.

[60] Huang QC (2007). Two thousand years of Guangdong's opening foreign trade——around Guangzhou. Shenzhen Univ J.

[61] Lennon JT, Smith VH, Dzialowski AR (2003). Invasibility of plankton food webs along a trophic state gradient. Oikos..

[62] Mello N, Maia-Barbosa PM (2015). Cyanobacteria bloom: selective filter for zooplankton?. Braz J Biol.

[63] Jiang T, Liu Z, Chen X (2005). Assessment of reservoir eutrophication in Guangdong province. J Lake Sci.

[64] Lampert W (1994). Phenotypic plasticity of the filter screens in *Daphnia*: adaptation to a low-food environment. Limnol Oceanogr.

[65] Wu T (2001). The Qinghai–Tibetan plateau: how high do Tibetans live?. High Alt Med Biol.

[66] MacArthur RI, Wilson EO. The theory of island biogeography. New Jersey: Princeton Univ Press; 1967.

