# Supplementary material for: Diversity of the Bosmina (Cladocera: Bosminidae) in China, revealed by analysis of two genetic markers (mtDNA 16S and a nuclear ITS)
Source: BMC Evol Biol. 2019 Jul 16;19:145. doi: 10.1186/s12862-019-1474-4 (PMC6635994; doi:10.1186/s12862-019-1474-4)
Supplement: Supplementary file 1 — Table S1. Numbers of observed haplotypes (of the 16S and ITS) in the investigated Chinese waterbodies; Table S2. The waterbodies where Bosmina was not found; Table S3. List of GenBank reference clones from Japanese, North American, European, Southern and Central Asian specimens used in phylogenetic analyses and haplotype networks. (DOCX 27 kb) [file 12862_2019_1474_MOESM1_ESM.docx]

Table SI Numbers of observed haplotypes (of the 16S and ITS) in the investigated Chinese waterbodies.

| Waterbodies name (abbreviation) | Genetic analyze | | |
| --- | --- | --- | --- |
|  | 16S haplotype |  | ITS haplotype |
| Eastern Plain Region (EPR) |  |  |  |
| Chao Lake (CAH) | CNm1(7) CAHma(2) |  | CNn1(1) CNn2(7) CNn3(2) |
| Changjiang Reservoir (CJR) | CJRma (9) CJRmb (1) |  | CNn3(1) CJRna(6) |
| Dong Lake (DOH) | CNm7(1) |  | CNn2(2) CNn3(1) |
| Dongping Lake (DPH) | CNm3(3) CNm4(5) |  | CNn1(7) DPHna(3) |
| Dongting Lake (DTH) | CNm1(8) |  | CNn2(10) |
| Gaoyou Lake (GYH) | CNm1(8) |  | CNn2(10) |
| Hongze Lake (HZH) | CNm1(5) HZHma(2) HZHmb(1) |  | CNn2(9) |
| Lulun Reservoir (LHR) | CNm3(3) |  | CNn1(3) CNn5(3) |
| Luo Lake (LUH) | CNm3(6) CNm4(2) |  | CNn1(4) CNn5(6) |
| Liujiaxia Reservoir (LJXR) | CNm2(1) CNm4(6) CNm6(1) |  | CNn1(10) |
| Luoma Lake (LMH) | CNm1(8) |  | CNn2(9) |
| Poyang Lake (PYH) | CNm1(6) CNm7(1) PYHma(1) |  | CNn1(1) CNn2(6) CNn3(1) CNn4(1) |
| Qiandao Lake (QDH) | CNm1(6) QDHma(1) |  | CNn2(9) CNn4(1) |
| Songtao Reservoir (STR) | CNm1(8) |  | CNn2(10) |
| Tai Lake (TAH) | CNm1(7) TAHma(1) |  | CNn2(10) |
| Xiliang Lak (XLH) | CNm1(5) XLHma(1) |  | CNn2(8) CNn4(2) |
| Xinfengjiang Reservoir (XFJR) | CNm1(6) |  | CNn2(7) |
| Ying Lake (YIH) | CNm1(7) |  | CNn2(10) |
| Mongolia–Xinjiang Plateau (MXP) |  |  |  |
| Bositeng Lake (BSTH) | CNm2(2) CNm5(5) |  | CNn1(9) |
| Hulun Lake (HLH) | CNm1(3) |  | CNn1(1) CNn2(4) |
| Hasuhai Lake (HSH) | CNm1(3) CNm4(2) |  | CNn1(2) CNn2(3) |
| Kundulun Lake (KDLH) | CNm2(5) CNm3(5) |  | CNn1(10) |
| TianChi (TIC) | CNm2(2) CNm5(5) |  | CNn1(7) |
| Wulungu Lake (WLGH) | CNm5 (8) |  | CNn1(3) WLGHna(4) WLGHnb(2) WLGHnc(1) |
| Northeast China Region (NCP) |  |  |  |
| Chagan Lake (CGH) | CNm1(7) |  | CNn2(8) CGHna(2) |
| Erlongshan Reservoir (ELSR) | CNm1(6) CNm6(2) |  | CNn1(2) CNn2(8) |
| Songhua Lake (SHH) | CNm1(7) |  | CNn1(1) CNn2(9) |
| Yunnan–Guizhou Plateau (YGP) |  |  |  |
| Ahang Reservoir (AHR) | CNm1(9) |  | CNn2(10) |
| Changshou Lake (CSH) | CNm1(6) CNm6(2) |  | CNn1(3) CNn2(7) |
| Dianchi (DIC) | DICma (8) |  | CNn2(10) |
| Fuxian Lake (FXH) | CNm1(1) CNm4(2) |  | CNn1(2) CNn2(1) FXHna(7) |
| Hongfeng Lake (HFH) | CNm1(10) |  | CNn2(8) |
| Heilongtan Reservoir (HLTR) | CNm1(10) |  | CNn2(10) |
| Sancha Lake (SCR) | CNm1(9) |  | CNn2(7) CNn4(1) |
| Yecheng Lake (YCH) | CNm1(1) |  | CNn1(2) CNn2(1) |

CNm, different waterbodies share the same 16S haplotype; CNn, different waterbodies share the same ITS haplotype; others with abbreviation of waterbody are private haplotype of corresponding waterbody respectively. a number in brackets indicates number of individuals possessing the identical sequence per waterbody.

Table SII The waterbodies where Bosmina were not found.

| Waterbodies name (abbreviation) | Latitude  (N) | Longitude(E) | Altitude(m) | WT (℃) | SD (m) | TP  (μg L^−1^) | Chl. a  (mg L^−1^) | T.S.I. | Trophic level |
| --- | --- | --- | --- | --- | --- | --- | --- | --- | --- |
| Eastern Plain Region (EPR) |  |  |  |  |  |  |  |  |  |
| Dawangtan Reservoir (DWTR) | 22.35 | 108.18 | 246 | 26.7±0.41 | 0.74±1.16 | 82.02±2.58 | 3.06±0.58 | 61.23 | Eutro |
| Nan Lake (NAH) | 29.2 | 113.06 | 186 | 26.8±2.68 | 0.8±1.62 | 73.45±0.16 | 2.80±0.72 | 59.76 | Eutro |
| Northeast China Region (NCP) |  |  |  |  |  |  |  |  |  |
| Wudalianchi (WDLC) | 48.44 | 126.11 | 513 | 26.6±4.48 | 0.65±1.70 | 56.24±0.96 | 1.02±0.23 | 42.59 | Oligo |
| Qinghai–Tibet Plateau (QTP) |  |  |  |  |  |  |  |  |  |
| Bitahai (BTH) | 27.83 | 99.98 | 3538 | 18±3.08 | 1.5±1.70 | 11.02±2.22 | 1.10±3.21 | 38.97 | Oligo |
| Cuona Lake (CNH) | 33.82 | 92.2 | 4650 | 18.7±3.50 | 1.65±0.24 | 17.39±85.07 | 0.83±19.20 | 38.32 | Oligo |
| Cuoguo Lake (CUGH) | 36.63 | 100.72 | 4313 | 20.8±3.91 | 1.62±1.65 | 11.85±3.12 | 0.77±0.30 | 40.09 | Oligo |
| Duobu Reservoir (DBH) | 28.93 | 87.08 | 3976 | 21.5±3.31 | 1.9±1.05 | 10.19±0.37 | 1.08±0.77 | 38.58 | Oligo |
| Nianchu River (NCH) | 29.28 | 88.9 | 4200 | 22.6±3.65 | 2.92±0.84 | 8.19±1.12 | 1.16±0.60 | 38.25 | Oligo |
| Namucuo (NMC) | 30.74 | 90.58 | 4718 | 21.6±2.63 | 1.46±0.20 | 11.32±1.72 | 1.11±0.74 | 40.01 | Oligo |
| Niyangqu (NYQ) | 29.67 | 94.34 | 5135 | 20.6±4.74 | 1. 56±1.10 | 17.41±0.88 | 0.86±1.83 | 38.59 | Oligo |
| Yang Lake (YAH) | 29.22 | 90.62 | 4441 | 19.5±4.85 | 1.65±4.62 | 11.90±4.06 | 0.79±2.38 | 41.01 | Oligo |
| Yigong Lake (YGH) | 30.3 | 94.86 | 2600 | 19.8±2.24 | 2.0±0.70 | 12.19±2.88 | 1.08±0.85 | 42.58 | Oligo |
| ZhikongdianzhanReservoir (ZKDZR) | 29.99 | 91.88 | 3888 | 20.9±5.28 | 1.62±0.38 | 20.00±4.10 | 0.86±0.37 | 43.17 | Oligo |

Table SIII List of GenBank reference clones from Japanese, North American, European, Southern and Central Asian specimens used in phylogenetic analyses and haplotype networks.

| Species name | Geographic name (abbreviation) | GenBank accession number (Haplotype abbreviations) | |
| --- | --- | --- | --- |
|  |  | 16S | ITS |
| *B. longirostris* | Salmon Lake, AK, USA (SAL) | EU650708 (AKm2) |  |
| *B. longirostris* | Alder Pond, AK, USA (ALP) | AF484005 (AKm3) | AF482744 (AKn3) |
| *B. longirostris* | Bagsværd Sø, Denmark (BAS) | EU650689 (DKm1) |  |
| *B. longirostris* | Großer Ploner See, Germany (GPS) | AF484003 (GERm4) | AF482743 (GERn4) |
| *B. longirostris* | Lake Aoki, Japan (LAO) | EU650690 (JPNm10) |  |
| *B. longirostris* | Lake Shikotsu, Japan (LSH) | EU650685 (JPNm11) |  |
| *B. longirostris* | Yomomarushi Ike Pond, Japan (YIP) | EU650707 (JPNm2) |  |
| *B. longirostris* | Watauchi Ike Pond, Japan (WIP) | EU650706 (JPNm3B) |  |
| *B. longirostris* | Lake Yamanaka, Japan (LYA) | EU650704 (JPNm4) |  |
| *B. longirostris* | Ichiyanagi Numa Pond, Japan (INP) | EU650703 (JPNm5) | EU650750 (JPNn5a) EU650748 (JPNn5b) |
|  |  |  | EU650752 (JPNn5c) EU650759 (JPNn5d) |
| *B. longirostris* | Lake Otori-Ike, Japan (LOI) | EU650702 (JPNm6) | EU650753 (JPNn6) |
| *B. longirostris* | Sainokami Ike Pond, Japan (SIP) | EU650701 (JPNm7) |  |
| *B. longirostris* | Onuma Pond in Onuma Quasi-National Park, Japan | EU650700 (JPNm8) | EU650751 (JPNn8a) EU650753 (JPNn8b) |
|  | (ONP) |  | EU650756 (JPNn8c) EU650760 (JPNn8d) |
| *B. longirostris* | Lake Ashi, Japan (LAS) | EU650688 (JPNm9B) |  |
| *B. longirostris* | Retaining pond, East Amherst, NY, USA (REP) | AF484004 (NYm9) |  |
| *B. longirostris* | Glubokoe Lake, Russia (GLL) | EU650686 (RUSm4A) |  |
| *B. fatalis* | Lake Suwa, Japan (LSU) | AF484009 (JPNm1) | AF482745 (JPNn1) |
| *B. hagmanni* | Gull Pond, MA, USA (GUP) | EU650725 (MAm4) |  |
| *B. hagmanni* | Great Pond, MA, USA (GRP) | EU650729 (MAm5) |  |
| *B. hagmanni* | Halls Pond, CT, USA (HAP) | AF484021 (MAm6) | AF482750 (MAn6) |
| *B. hagmanni* | Sunapee Lake, NH, USA (SUL) | EU650724 (NHm2) |  |
| *B. hagmanni* | Swan Pond, NY, USA (SWP) | EU650726 (NYm6) |  |
| *B. hagmanni* | North Dike Reservoir, SC, USA (NDR) | AF484019 (SCm1) | AF482749 (SCn1) |
| *B. hagmanni* | Swamp near Bamburg, SC, USA (SNB) | EY650727 (SCm2) |  |
| *B. hagmanni* | Paris Reservoir, AR, USA (PAR) | EU650728 (ARm2) |  |
| *B. tubicen* | Ril Lake, ON, Canada (RIL) | AF484020 (ONm6) | AF482751 (ONn6) |
| *B. coregoni* | Kuhn Lake, IN, USA (KUL) | AF484011 (INm1) | AF482756 (INn1) |
| *B. freyi* | Unnamed pond, Princeton, AR, USA (UPP) | AF484000 (ARm1) | AF482736 (ARn1) |
| *B. liederi* | Unnamed pond near Solomon, AK, USA (UPS) | AF484008 (AKm1) | AF482739 (AKn1) |
| *B. oriens* | Fresh Pond, Massachusetts, USA (FPM) | AF484018 (MAm8) | AF482746 (MAn8) |
| *B. longispina* | Unnamed pond #19 on Teller highway, AK, USA (UPT) | AY264737 (AKm4) | AY264768 (AKn4) |
| *B. tanakai* | Ichiyanagi Numa Pond, Japan (INP) | EU650733 (JPNm15b) | EU650766 (JPNn15a) |
