# Supplementary material for: Diversity of the Bosmina (Cladocera: Bosminidae) in China, revealed by analysis of two genetic markers (mtDNA 16S and a nuclear ITS)
Source: BMC Evol Biol. 2019 Jul 16;19:145. doi: 10.1186/s12862-019-1474-4 (PMC6635994; doi:10.1186/s12862-019-1474-4)
Supplement: Supplementary file 2 — The obtained haplotypes of 16S and ITS in this study. (DOCX 16 kb) [file 12862_2019_1474_MOESM2_ESM.docx]

**16S Haplotypes:**

>CNm1

AATAGCCGCAGTATTTTGACTGTGCTAAGGTAGCATAATCATTAGTCTTTTAATTGAAGGCTGGTATGAATGGCAAGACGAGAAAGAAGCTGTCTCTTTTAAAAAATTGAATTTCATTTTTAAGTGAAAAAGCTTAAATGTATTTAAGGGACGATCAGACCCTATGGAGCTTTATTTCGTTGATTAGTTAAATTTATATTTTAAAGTTAATTATTTAACGAAATTTTGTTGGGGCGACAAAGAGATAAAAATAACACTCTTTTTATTAAACATAAATAAATGAATAATTGATCCCTAAAGAAGGATTAAAAGACTAAGTTACCCTAGGGATAACAGCGTAATTTTTTTGGAGAGTTCAAATCGATAAAAAAGTTTGCGACCTCGATGTTGGATTAGGAATTTAACTAGGTGCAGAAGTTTAGTTGTAGAGTCTGTTCGACTTTTAAAATCCTACATGATCT

>CNm2

AATAGCCGCAGTATTTTGACTGTGCTAAGGTAGCATAATCATTAGTCTTTTAATTGAAGGCTGGTATGAATGGCAAGACGAGAAAGAAGCTGTCTCTCTAAAGATAATCGAATTTCATTTTTAAGTGAAAAAGCTTAAATAAATTTAAGGGACGATCAGACCCTATGGAGCTTAATTTTAAGATAAAAGTAAATTTATTTTTAAAAGTTACTTATTTTAAAAAATTTTGTTGGGGCGACAGAGAGATGAAAAAACCACTCTTTTTATTTAACTTATATTAAAGGAGAGCTGATCCCTAAAGAAGGATTAAAAGACTAAGTTACCCTAGGGATAACAGCGTAATTTTTTTGGAGAGTTCATATCGATAAAAAAGTTTGCGACCTCGATGTTGGATTAGGAATTTAACTAGGTGCAGAAGTTTAGTTGTAGAGTCTGTTCGACTTTTAAAATCCTACATGATCT

>CNm3

AATAGCCGCAGTATTTTGACTGTGCTAAGGTAGCATAATCATTAGTCTTTTAATTGAAGGCTGGTATGAATGGCAAGACGAGAAAGAAGCTGTCTCTTTGAAAATAATCGAATTTCATTTTTAAGTGAAAAAGCTTAAATAAATTTGAGGGACGATCAGACCCTATGGAGCTTAATTTTAAGATAAAAGTAAATTTATTTTTAAAAGTTACTTTTCTTAAAAAATTTTGTTGGGGCGACAGAGAGATGAAAAAACCACTCTTTTTTATTAAACTTATATTAAAGGAAAACTGATCCCTAAAGAAGGATTAAAAGACTAAGTTACCCTAGGGATAACAGCGTAATTTTTTTGGAGAGTTCATATCGATAAAAAAGTTTGCGACCTCGATGTTGGATTAGGAATTTAACTAGGTGCAGAAGTTTAGTTGTAGAGTCTGTTCGACTTTTAAAATCCTACATGATCT

>CAHma

AATAGCCGCAGTATTTTGACTGTGCTAAGGTAGCATAATCATTAGTCTTTTAATTGAAGGCTGGTATGAATGGCAAGACGAGAAAGTAGCTGTCTCTTTTAAAAAAATTGAATTTCATTTTTAAGTGAAAAAGCTTAAATATATTTAAGGGACGATCAGACCCTATGGAGCTTAATTTCATTAAATAGTTAAATTTATATTTAAAAGTTCACTGTTTAATAAAATTTTGTTGGGGCGACAAAGAGATAAAAATAACACTCTTTTTATTAAACACATATAGATGAAGAGATGATCCCTAAAGAAGGATTAAAAGACTAAGTTACCCTAGGGATAACAGCGTAATTTTTTTGGAGAGTTCAAATCGATAAAAAAGTTTGCGACCTCGATGTTGGATTAGGAATTTAACTAGGTGCAGAAGTTTAGTTGTAGAGTCTGTTCGACTTTTAAAATCCTACATGATCT

>DICma

AATAGCCGCAGTATTTTGACTGTGCTAAGGTAGCATAATCATTAGTCTTTTAATTGAAGGCTGGTATGAATGGCAAAACGAGAAAGAAGCTGTCTCTTTTAAAAAAATTGAATTTCATTTTTAAGTGAAAAAGCTTAAATGTATTTAAGGGACGATCAGACCCTATGGAGCTTTATTTCGTTGATTAGTTAAATTTATATTTTAAAGTTAATTATTTAACGAAATTTTGTTGGGGCGACAAAGAGATAAAAATAACACTCTTTTTATTAAACATAAATAAATGAATAATTGATCCCTAAAGAAGGATTAAAAGACTAAGTTACCCTAGGGATAACAGCGTAATTTTTTTGGAGAGTTCAAATCGATAAAAAAGTTTGCGACCTCGATGTTGGATTAGGAATTTAACTAGGTGCAGAAGTTTAGTTGTAGAGTCTGTTCGACTTTTAAAATCCCACATGATCT

>CNm4

AATAGCCGCAGTATTTTGACTGTGCTAAGGTAGCATAATCATTAGTCTTTTAATTGAAGGCTGGTATGAATGGCAAGACGAGAAAGAAGCTGTCTCTTTGAAAATAATCGAATTTCATTTTTAAGTGAAAAAGCTTAAATAAATTTGAGGGACGATCAGACCCTATGGAGCTTAATTTTAAGATAAAAGTAAATTTATTTTTAAAAGTTACTTTTCTTAAAAAATTTTGTTGGGGCGACAGAGAGATGAAAAAATCACTCTTTTTATTAAACTTATATTAAAGGAAAATTGATCCCTAAAGAAGGATTAAAAGACTAAGTTACCCTAGGGATAACAGCGTAATTTTTTTGGAGAGTTCATATCGATAAAAAAGTTTGCGACCTCGATGTTGGATTAGGAATTTAACTAGGTGCAGAAGTTTAGTTGTAGAGTCTGTTCGACTTTTAAAATCCTACATGATCT

>HZHma

AATAGCCGCAGTATTTTGACTGTGCTAAGGTAGCATAATCATTAGTCTTTTAATTGAAGGCTGGTATGAATGGCAAGACGAGAAAGAAGCTGTCTCTTTTAAAAAAATTGAATTTCATTTTTAAGTGAAAAAGCTTAAATGTGTTTAAGGGACGATCAGACCCTATGGAGCTTTATTTCGTTGATTAGTTAAATTTATATTTTAAAGTTAATTATTTAACGAAATTTTGTTGGGGCGACAAAGAGATAAAAATAACACTCTTTTTATTAAACATAAATAAATGAATAATTGATCCCTAAAGAAGGATTAAAAGACTAAGTTACCCTAGGGATAACAGCGTAATTTTTTTGGAGAGTTCAAATCGATAAAAAAGTTTGCGACCTCGATGTTGGATTAGGAATTTAACTAGGTGCAGAAGTTTAGTTGTAGAGTCTGTTCGACTTTTAAAATCCTACATGATCT

>CNm5

AATAGCCGCAGTATTTTGACTGTGCTAAGGTAGCATAATCATTAGTCTTTTAATTGAAGGCTGGTATGAATGGCAAGACGAGAAAGAAGCTGTCTCTTTAAAATTAATTGAATTTCATTTTTAAGTGAAAAAGCTTAAATAAGTTTAAGGGACGATCAGACCCTATGGAGCTTAATTTTAATTTGTGAGTAAATTTATTTTTAAAAGTTACTTGCTTTAAAAAATTTTGTTGGGGCGACAGAGAGATGAAAAAACCACTCTTTTTATTAAACTTATGTTAATGGAAAATTGATCCCTAAAGAAGGATTAAAAGACTAAGTTACCCTAGGGATAACAGCGTAATTTTTTTGGAGAGTTCATATCGATAAAAAAGTTTGCGACCTCGATGTTGGATTAGGAATTTAGCTAGGCGCAGAAGTTTAGTTGTAGAGTCTGTTCGACTTTTAAAATCCTACATGATCT

>CNm6

AATAGCCGCAGTATTTTGACTGTGCTAAGGTAGCATAATCATTAGTCTTTTAATTGAAGGCTGGTATGAATGGCAAGACGAGAAAGAAGCTGTCTCTTTAAAAAGAATCGAATTTCATTTTTAAGTGAAAAAGCTTAAATAAATTTGAGGGACGATCAGACCCTATGGAGCTTAATTTTAAGATAAAAGCAAATTTATTTTTAAAAGTTACTTATTTTAAAAAATTTTGTTGGGGCGACAGAGAGATGAAAAAACCACTCTTTTTATTAAACTTATGTTAAAGGAGAATTGATCCCTAAAGAAGGATTAAAAGACTAAGTTACCCTAGGGATAACAGCGTAATTTTTTTGGAGAGTTCATATCGATAAAAAAGTTTGCGACCTCGATGTTGGATTAGGAATTTAACTAGGTGCAGAAGTTTAGTTGTAGAGTCTGTTCGACTTTTAAAATCCTACATGATCT

>CNm7

AATAGCCGCAGTATTTTGACTGTGCTAAGGTAGCATAATCATTAGTCTTTTAATTGAAGGCTGGTATGAATGGCAAGACGAGAAAGTAGCTGTCTCTTTTAAAAAAATTGAATTTCATTTTTAAGTGAAAAAGCTTAAATATATTTAAGGGACGATCAGACCCTATGGAGCTTAATTTTATTAAATAGTTAAATTTATATTTAAAAGTTCACTGTTTAATAAAATTTTGTTGGGGCGACAAAGAGATAAAAATAACACTCTTTTTATTAAACACATATAGATGAAGAGATGATCCCTAAAGAAGGATTAAAAGACTAAGTTACCCTAGGGATAACAGCGTAATTTTTTTGGAGAGTTCAAATCGATAAAAAAGTTTGCGACCTCGATGTTGGATTAGGAATTTAACTAGGTGCAGAAGTTTAGTTGTAGAGTCTGTTCGACTTTTAAAATCCTACATGATCT

>HZHmb

AATAGCCGCAGTATTTTGACTGTGCTAAGGTAGCATAATCATTAGTCTTTTAATTGAAGGCTGGTATGAATGGCAAGACGAGAAAGAAGCTGTCTCTTTTAAAAAAATTGAATTTCATTTTTAAGTGAAAAAGCTTAAATGTATTTAAGGGACGATCAGACCCTATGGAGCTTTATTTCGTTGATTAGTTAAATTTATATTTTAAAGTTAATTATTTGACGAAATTTTGTTGGGGCGACAAAGAGATAAAAATAACACTCTTTTTATTAAACATAAATAAATGAATAATTGATCCCTAAAGAAGGATTAAAAGACTAAGTTACCCTAGGGATAACAGCGTAATTTTTTTGGAGAGTTCAAATCGATAAAAAAGTTTGCGACCTCGATGTTGGATTAGGAATTTAACTAGGTGCAGAAGTTTAGTTGTAGAGTCTGTTCGACTTTTAAAATCCTACATGATCT

>PYHma

AATAGCCGCAGTATTTTGACTGTGCTAAGGTAGCATAATCATTAGTCTTTTAATTGAAGGCTGGTATGAATGGCAAGACGAGAAAGAAGCTGTCTCTTTAAAAAGAATCGAATTTCATTTTTAAGTGAAAAAGCTTAAATAAATTTGAGGGACGATCAGACCCTATGGAGCTTAATTTTAAGATAAAAAGCAAATTTATTTTTAAAAGTTACTTATTTTAAAAAATTTTGTTGGGGCGACAGAGAGATGAAAAAACCACTCTTTTTATTAAACTTATATTAAAGGAGAGTTGATCCCTAAAGAAGGATTAAAAGACTAAGTTACCCTAGGGATAACAGCGTAATTTTTTTGGAGAGTTCATATCGATAAAAAAGTTTGCGACCTCGATGTTGGATTAGGAATTTAACTAGGTGCAGAAGTTTAGTTGTAGAGTCTGTTCGACTTTTAAAATCCTACATGATCT

>QDHma

AATAGCCGCAGTATTTTGACTGTGCTAAGGTAGCATAATCATTAGTCTTTTAATTGAAGGCTGGTATGAATGGCAAGACGAGAAAGAAGCTGTCTCTTTTAAAAAAATTGAATTTCATTTTTAAGTGAAAAAGCTTAAATGTATTTAAGGGACGATCAGACCCTATGGAGCTTTATTTCGTTGCTTAGTTAAATTTATATTTTAAAGTTAATTATTTAACGAAATTTTGTTGGGGCGACAAAGAGATAAAAATAACACTCTTTTTATTAAACATAAATAAATGAATAATTGATCCCTAAAGAAGGATTAAAAGACTAAGTTACCCTAGGGATAACAGCGTAATTTTTTTGGAGAGTTCAAATCGATAAAAAAGTTTGCGACCTCGATGTTGGATTAGGAATTTAACTAGGTGCAGAAGTTTAGTTGTAGAGTCTGTTCGACTTTTAAAATCCTACATGATCT

>CJRma

AATAGCCGCAGTATTTTGACTGTGCTAAGGTAGCATAATCATTAGTCTCTTAATTGGAGGCTGGTATGAATGGCAAGACGAGAAAGAAACTGTCTCTAAGAAAAATATTGAAATTCATTTTTTAGTGAAAAAGCTAAAATGTATTCAAGGGACGATCAGACCCTATGGAGCTTTATTACTTTGAGAATTCAAATTTATAAAAATAGATTTTTCATTAAATTTTGTTGGGGCGACAAAGAGTTTTAAGTAACACTCTTTTTTAAAAACATACATAAATGGAAAAAGGATCCCCAGAGAGGGATTACAAGACTAAGTTACCCTAGGGATAACAGCGTAATTTTTTTGGAGAGTTCTTATCGATAAAAAAGTTTGCGACCTCGATGTTGGATTAGGAATTTAGCTAGGTGCAGAAGTTTAGCTGTAGAGTCTGTTCGACTTT-AAAATCCTACATGATCT

>CJRmb

AATAGCCGCAGTATTTTGACTGTGCTAAGGTAGCATAATCATTAGTCTTTTAATTGAAGGCTGGTATGAATGGCAAGACGAGAAAGTAGCTGTCTCTTTTAAAAAAATTGAATTTCATTTTTAAGTGAAAAAGCTTAAATACATTTAAGGGACGATCAGACCCTATGGAGCTTAATTTTATTAAACAGTTAAATTTATATTTGAAAGTTCACTGTTTAATAAAATTTTGTTGGGGCGACAAAGAGATAAAAATAACACTCTTTTTATTAAACACATATAGATGAAGAGATGATCCCTAAAGAAGGATTAAAAGACTAAGTTACCCTAGGGATAACAGCGTAATTTTTTTGGAGAGTTCAAATCGATAAAAAAGTTTGCGACCTCGATGTTGGATTAGGAATTTAACTAGGTGCAGAAGTTTAGTTGTAGAGTCTGTTCGACTTTTAAAATCCTACATGATCT

>TAHma

AATAGCCGCAGTATTTTGACTGTGCTAAGGTAGCATAATCATTAGTCTTTTAATTGAAGGCTGGTATGAATGGCAAGACGAGAAAGAAGCTGTCTCTTTTAAAAAATTGAATTTCATTTTTAAGTGAAAAAGCTTAAATGTATTTAAGGGACGATCAGACCCTATGGAGCTTTATTTCGTTGATTAGTTAAATTTATATTTTAAAGTTAAATATTTAACGAAATTTTGTTGGGGCGACAAAGAGATAAAAATAACACTCTTTTTATTAAACATAAATAAATGAATAATTGATCCCTAAAGAAGGATTAAAAGACTAAGTTACCCTAGGGATAACAGCGTAATTTTTTTGGAGAGTTCAAATCGATAAAAAAGTTTGCGACCTCGATGTTGGATTAGGAATTTAACTAGGTGCAGAAGTTTAGTTGTAGAGTCTGTTCGACTTTTAAAATCCTACATGATCT

>XLHma

AATAGCCGCAGTATTTTGACTGTGCTAAGGTAGCATAATCATTAGTCTTTTAATTGAAGGCTGGTATGAATGGCAAGACGAGAAAGAAGCTGTCTCTTTTAAAAAAATTGAATTTCATTTTTAAGTGAAAAAGCTTAAATGTATTTAAGGGACGATCAGACCCTATGGAGCTTTATTTTGTTGTTTAGTTAAATTTATATTTTAAAGTTAATTATTTAACGAAATTTTGTTGGGGCGACAAAGAGATAAAAATAACACTCTTTTTATTAAACATAAATAAATGAATAATTGATCCCTAAAGAAGGATTAAAAGACTAAGTTACCCTAGGGATAACAGCGTAATTTTTTTGGAGAGTTCAAATCGATAAAAAAGTTTGCGACCTCGATGTTGGATTAGGAATTTAACTAGGTGCAGAAGTTTAGTTGTAGAGTCTGTTCGACTTTTAAAATCCTACATGATCT

**ITS Haplotypes:**

>CNn1

GTTCAAACTTGATCCTTTAGAGGAAGTAAAAGTCGTAACAAGGTTTCCGTAGGTGAACCTGCGGAAGGATCATAATCGATGAACATGTACCGTTCTTAAGCGCCCACAAGCCCTTAGACGAGTATGTATTTCTTAAAGCCTTATACGGGCGAAATAAAGTCAATGTGAAGGAGACGGGTACCAGGACGCTACGTCAAGGTTCAAAGAAATTCGCCCGACTTCTCTCACAGCACACACAAACCTCTAGTAACCACGGTAGTTGGAAAACATTCGAAACGCCATAGTCTTTGCGAAAGCAAAGACAAAAAGCGCTCGAAGAACACAATGTGTCATAATTATGACCCTGAACGGTGGATCACTCGGCTCGTGGATCGATGAAGAGCGCAGCAAAGTGCGCTAATCCATGCGAACCGCAGAACACATGGAGCATCGAAATCTTGAACGTAAATGGCGGCCCAGCTTCACTGCTCGGGCCACATCTGACTGAGGGTCGGTTGATTGATGAACGATTATTCTGGGTGCCGAGCGGAGAGAAATTTCCATCGATGCCTTAAATTCAATCGGTCAACACTCGATGGGAGGCATTCTTAAGAGTCTGAACGGCACCGAAGTGTCTGGAGCCCAAACGACTCTTATTGCT-ACAAACCCTGTCGGTGTTTGTGACCGTAAAAGCTAAGTACAGACTTAAGTTTGCTCATAAGGCGTTTGAGGCAGTGTAAAGTCTCGGACGGTACGTGTGTGTTTGTCTGTACGCCGGGCGTGTCGGGAAACTGACCACCGCGATGAGACAGCCGTACCAAGACAAATCACTGACGAGCCTGTGAGTAGCTCAAGTCGATACAAGTACAGAGCGCTAATCTAATCATATCTGACCTCAGTTTAGGTGAGACTACCCGCTGAACTTAAGCATATCAGTAAGCGGAGGAAAAGAAACTAACAAGGATTCCCTTAGTAGCGGCGAGCGAACAGGGAAGAGCCCAGCACCGAATCCCGCGCCCTGTAAAGAGGACGCAGGGAAATGTGGTGTTTGGGAGGGCCGTGCGCGCCTGTCCGGTATTAGCCCAAGTTCTCATGAACGAGACGAGCAACCCATAGAGGGTGTCAGGCCCGTCGATAGCTAGCCGCGACCGTGCAT

> WLGHna

GTTCAAACTTGATCCTTTAGAGGAAGTAAAAGTCGTAACAAGGTTTCCGTAGGTGAACCTGCGGAAGGATCATAATCGATGAACATGTACCGTTCTTAAGCGCCCACAAGCCCTTAGACGAGTATGTATTTCTTAAAGCCTTATACGGGCGAAATAAAGTCAATGTGAAGGAGACGGGTACCAGGACGCTACGTCAAGGTTCAAAGAAATTCGCCCGACTTCTCTCACAGCACACACAAACCTCTAGTAACCACGGTAGTTGGAAAACATTCGATACGCCATAGTCTTTGCGAAAGCAAAGACAAAAAGCGCTCGAAGAACACAATGTGTCATAATTATGACCCTGAACGGTGGATCACTCGGCTCGTGGATCGATGAAGAGCGCAGCAAAGTGCGCTAATCCATGCGAACCGCAGAACACATGGAGCATCGAAATCTTGAACGTAAATGGCGGCCCAGCTTCACTGCTCGGGCCACATCTGACTGAGGGTCGGTTGATTGATGAACGATTATTCTGGGTGCCGAGCGGAGAGAAATTTCCATCGATGCCTTAAATTCAATCGGTCAACACTCGATGGGAGGCATTCTTAAGAGTCTGAACGGCACCGAAGTGTCTGGAGCCCAAACGACTCTTATTGCTACAAACCCTGTCGGTGTTTGTGACCGTAAAAGCTAAGTACAGACTTAAGTTTGCTCATAAGGCGTTTGAGGCAGTGTAAAGTCTCGGACGATACGTGTGTGTTTGTCTGTACGCCGGGCGTGTCGGGAAACTGACCACCGCGATGAGACAGCCGTACCAAGACAAATCACTGACGAGCCTGTGAGTAGCTCAAGTCGATACAAGTACAGAGCGCTAATCTAATCATATCTGACCTCAGTTTAGGTGAGACTACCCGCTGAACTTAAGCATATCAGTAAGCGGAGGAAAAGAAACTAACAAGGATTCCCTTAGTAGCGGCGAGCGAACAGGGAAGAGCCCAGCACCGAATCCCGCGCCCTGTAAAGAGGACGCAGGGAAATGTGGTGTTTGGGAGGGCCGTGCGCGCCTGTCCGGTATTAGCCCAAGTTCTCATGAACGAGACGAGCAACCCATAGAGGGTGTCAGGCCCGTCGATAGCTAGCCGCGACCGTGCAT

> WLGHnb

GTTCAAACTTGATCCTTTAGAGGAAGTAAAAGTCGTAACAAGGTTTCCGTAGGTGAACCTGCGGAAGGATCATAATCGATGAACATGTACCGTTCTTAAGCGCCCACAAGCCCTTAGACGAGTATGTATTTCTTAAAGCCTTATACGGGCGAAATAAAGTCAATGTGAAGGAGACGGGTACCAGGACGCTACGTCAAGGTTCAAAGAAATTCGCCCGACTTCTCTCACAGCACACACAAACCTCTAGTAACCACGGTAGTTGGAAAACATTCGATACGCCATAGTCTTTGCGAAAGCAAAGACAAAAAGCGCTCGAAGAACACAATGTGTCATAATTATGACCCTGAACGGTGGATCACTCGGCTCGTGGATCGATGAAGAGCGCAGCAAAGTGCGCTAATCCATGCGAACCGCAGAACACATGGAGCATCGAAATCTTGAACGTAAATGGCGGCCCAGCTTCACTGCTCGGGCCACATCTGACTGAGGGTCGGTTGATTGATGAACGATTATTCTGGGTGCCGAGCGGAGAGAAATTTCCATCGATGCCTTAAATTCAATCGGTCAACACTCGATGGGAGGCATTCTTAAGAGTCTGAACGGCACCGAAGTGTCTGGAGCCCAAACGACTCTTATTGCTACAAACCCTGTCGGTGTTTGTGACCGTAAAAGCTAAGTACAGACTTAAGTTTGCTCATAAGGCGTTTGAGGCAGTGTAAAGTCTCGGACGGTACGTGTGTGTTTGTCTGTACGCCGGGCGTGTCGGGAAACTGACCACCGCGATGAGACAGCCGTACCAAGACAAATCACTGACGAGCCTGTGAGTAGCTCAAGTCGATACAAGTACAGAGCGCTAATCTAATCATATCTGACCTCAGTTTAGGTGAGACTACCCGCTGAACTTAAGCATATCAGTAAGCGGAGGAAAAGAAACTAACAAGGATTCCCTTAGTAGCGGCGAGCGAACAGGGAAGAGCCCAGCACCGAATCCCGCGCCCTGTAAAGAGGACGCAGGGAAATGTGGTGTTTGGGAGGGCCGTGCGCGCCTGTCCGGTATTAGCCCAAGTTCTCATGAACGAGACGAGCAACCCATAGAGGGTGTCAGGCCCGTCGATAGCTAGCCGCGACCGTGCAT

> CNn2

GTTCAAACTTGATCCTTTAGAGGAAGTAAAAGTCGTAACAAGGTTTCCGTAGGTGAACCTGCGGAAGGATCATAATCGAATGAACATGTACAGTTCTAAAGCGCCTACAAGCCTTTAGACGAGTATATATTTATAGAAGCCTTTAACAGGCGAAATAAAGTCAATGTGAAGGAGACGGGTACCAGGACGTTAGTCAAGGTTCAAAGAATTTCGCCCGACTTCTCTCACAGCACACACAAACCTCTAGTAACCACGGTAGTTGGAAAATATTCGAAGCGTCGTAGTCTTGGCGAAAGCAAAGACGAAAAGCGCTCGAAGAACACAAAGTGTCATAATTATGACCCTGAACGGTGGATCACTCGGCTCGTGGATCGATGAAGAGCGCAGCAAAGTGCGCTAATCCATGCGAACCGCAGAACACATGGAGCATCGAAATCTTGAACGTAAATGGCGGCCCAGCTTCACTGCTCGGGCCACATCTGACTGAGGGTCGGTTGATTGATGAACGATTATTCTGGGTGCCGAGCGGAGAGAAATTTCCATCGATGCCTTAAATTCAATCGGTCAACACTCGATGGGAGGCATTCGTAAGAGTCTGAGCGGCACCGAAGTGTCTGGAGCCCAAACGAATCTTGCTTGCTATCAACCCTGTCGGTGTTTGTGACTGTAAATGCTAAGTACAGACTCAAGTTTGCTCATAAGGCGTTTGAGGCAGTGTATAGTCTCGGACGGTACGTGTGTGTTTGTCTGTACGCCGGGCGTGTCGGGAAACTGACCACCGCGATGAGACAGCCGTACCAAGACAAATCACTGGCGAGCCTGTGAGTAGCTCAAGTCGATACAAGTACAAAGCGCTAATCTAATCATATCTGACCTCAGTTTAGGTGAGACTACCCGCTGAACTTAAGCATATCAGTAAGCGGAGGAAAAGAAACTAACAAGGATTCCCTCAGTAGCGGCGAGCGAACAGGGAAGAGCCCAGCACCGAATCCCGCGCCCTGCAAAGAGGACGCAGGGAAATGTGGTGTTTGGGAGGGCCGTGCGCGCCTGTCCGGTATTAGCCCAAGTTCTCATGAACGAGACGAGCAACCCATAGAGGGTGTCAGGCCCGTCGATAGCTAGCCGCGACCGTGCAT

> CNn3

GTTCAAACTTGATCCTTTAGAGGAAGTAAAAGTCGTAACAAGGTTTCCGTAGGTGAACCTGCGGAAGGATCATAATCGAATGAACATATACAGTTCTAAAGCGCCTACAAGCCTTTAGACGAGTATATATTTCTAAAAGCCTTTAACAGGCGAAATAAAGTCAATGTGAAGGAGACGGGTACCAGGACGTTAGTCAAGGTTCAAAGAATTTCGCCCGACTTCTCTCACAGCACACACAAACCTCTAGTAACCACGGTAGTTGGAAAATATTCGAAACGTTGTAGTCTTGGCGAAAGCAAAGACGAGAAGCGCTCGAAAAACACAATGTGTCATAATTATGACCCTGAACGGTGGATCACTCGGCTCGTGGATCGATGAAGAGCGCAGCAAAGTGCGCTAATCCATGCGAACCGCAGAACACATGGAGCATCGAAATCTTGAACGTAAATGGCGGCCCAGCTTCACTGCTCGGGCCACATCTGACTGAGGGTCGGTTGATTGATGAACGATTATTCTGGGTGCCGAACGGAGAGAAATTTCCATCGATGCCTTAAATTCAATCGGTCAACACTCGATGGGAGGCATTCTTAAGAGTCTGAGCGGCACCGAAGTGTCTGGAGCCCAATCGAATCTTATTGCTATCAACCCTGTCGGTGTTTGTGACTGTAAAAGCTAAGTACAGACTAAAGTTTGCTCATAAGGCGTTTGAGGCAGTGTATAGTCTCGGACGGTACGTGTGTGTTTGTCTGTACGCCGGGCGTGTCGGGAAACTGACCACCGCGATGAGACAGCCGTACCAAGACAAATCACTGACGAGCCTGTGAGTAGCTCAAGTCGATACAAGTACAAAGCGCTAATCTAATCATATCTGACCTCAGTTTAGGTGAGACTACCCGCTGAACTTAAGCATATCAGTAAGCGGAGGAAAAGAAACTAACAAGGATTCCCTCAGTAGCGGCGAGCGAACAGGGAAGAGCCCAGCACCGAATCCCGCGCCCTGCAAAGAGGACGCAGGGAAATGTGGTGTTTGGGAGGGCCGTGCGCGCCTGTCCGGTATTAGCCCAAGTTCTCATGAACGAGACGAGCAACCCATAGAGGGTGTCAGGCCCGTCGATAGCTAGCCGCGACCGTGCAT

> WLGHnc

GTTCAAACTTGATCCTTTAGAGGAAGTAAAAGTCGTAACAAGGTTTCCGTAGGTGAACCTGCGGAAGGATCATAATCGATGAACATGTACCGTTCTTAAGCGCCCACAAGCCCTTAGACGAGTATGTATTTCTTAAAGCCTTATACGGGCGAAATAAAGTCAATGTGAAGGAGACGGGTACCAGGACGCTACGTCAAGGTTCAAAGAAATTCGCCCGACTTCTCTCACAGCACACACAAGCCTCTAGTAACCACGGTAGTTGGAAAACATTCGAAACGCCATAGTCTTTGCGAAAGCAAAGACAAAAAGCGCTCGAAGAACACAATGTGTCATAATTATGACCCTGAACGGTGGATCACTCGGCTCGTGGATCGATGAAGAGCGCAGCAAAGTGCGCTAATCCATGCGAACCGCAGAACACATGGAGCATCGAAATCTTGAACGTAAATGGCGGCCCAGCTTCACTGCTCGGGCCACATCTGACTGAGGGTCGGTTTATTGATGAACGATTATTCTGGGTGCCGAGCGGAGAGAAATTTCCATCGATGCCTTAAATTCAATCGGTCAACACTCGATGGGAGGCATTCTTAAGAGTCTGAACGGCACCGAAGTGTCTGGAGCCCAAACGACTCTTATTGCTACAAACCCTGTCGGTGTTTGTGACCGTAAAAGCTAAGTACAGACTTAAGTTTGCTCATAAGGCGTTTGAGGCAGTGTAAAGTCTCGGACGGTACGTGTGTGTTTGTCTGTACGCCGGGCGTGTCGGGAAACTGACCACCGCGATGAGACAGCCGTACCAAGACAAATCACTGACGAGCCTGTGAGTAGCTCAAGTCGATACAAGTACAGAGCGCTAATCTAATCATATCTGACCTCAGTTTAGGTGAGACTACCCGCTGAACTTAAGCATATCAGTAAGCGGAGGAAAAGAAACTAACAAGGATTCCCTTAGTAGCGGCGAGCGAACAGGGAAGAGCCCAGCACCGAATCCCGCGCCCTGTAAAGAGGACGCAGGGAAATGTGGTGTTTGGGAGGGCCGTGCGCGCCTGTCCGGTATTAGCCCAAGTTCTCATGAACGAGACGAGCAACCCATAGAGGGTGTCAGGCCCGTCGATAGCTAGCCGCGACCGTGCAT

> CJRna

GTTCAAACTTGATCCTTTAGAGGAAGTAAAAGTCGTAACAAGGTTTCCGTAGGTGAACCTGCGGAAGGATCATAATCGATAGACATATACCGTTCAAAACCGGCTGCAAGGCCTTTGACGAGTGTATATACATAAAAAAAAGCCCGTAAAAAGGCGAAATAAAGTCTATGTGAAGAAGACGGGTACCAAGAGGTTCAAAGCATTTCGCCCGATTTCTCTCACAGCACATTCAAAATATCTAGTATCCTCGGTAGTCGGAATATATTCGAAACGCTGAAAGTTTGTGCGAAAGCAAAGACCGTCAGCGCTCGAAGAACACAAAGTGTCATTATTATGACCCTGAACGGTGGATCACTCGGCTCGTGGATCGATGAAGAGCGCAGCAAAGTGCGCTAATCCATGCGAACCGCAGAACACATGGAGCATCGAAATCTTGAACGTAAATGGCGGCCCAGCTTCACTGCTCGGGCCACATCTGACTGAGGGTCGGTTGATTGATGAACGATTATTTTTGGGTGCCGAGCGGGAGGCAATTTCCCTCGATGCCTTAAATACAACTGGTCTACGATCGACGAAGGGCAACTATAGAGTCAAAGAAGTCGCACCGAAGTGTCTGGAGCTCTCACTTTGCATTCTTTATTGCTCGAAAACCTCGTCGTTCTTGAAAGACTGTGAATGCTAAGTACAGACTAAAAGTTTGCCCATAAGGCGTTTGAGGCAGTGTATAGTCTCGGACGGTATGTGTGTGTTTGTCTGTACGCCGGGCGTGTCGGGAAACTGACCACCGCGAAGAGACAGCCGTATCGAGACAAAACATTGACGAGCCTGTCGGTAGCTCGAGTCTCTAGAGTACGAAGCGCGAATATAATCATATCTGACCTCAGTTTAGGTGAGACTACCCGCTGAACTTAAGCATATCAGTAAGCGGAGGAAAAGAAACTAACAAGGATTCCCTTAGTAGCGGCGAGCGAACAGGGAAGAGCCCAGCACCGAATCCCGCGCCCTGCAAAGAGGACGCAGGGAAATGTGGTGTTTGGGAGGGCCGTGCGCGCCTGTCCGGTATTAGCCCAAGTTCTCATGAACGAGACGAGCAACCCGTAGAGGGTGTCAGGCCCGTCGATAGCTAGCCGTGACCGTGCAT

> CNn4

GTTCAAACTTGATCCTTTAGAGGAAGTAAAAGTCGTAACAAGGTTTCCGTAGGTGAACCTGCGGAAGGATCATAATCGAATGAACATGTACAGTTCTAAAGCGCCTACAAGCCTTTAGACGAGTATATATTTATAGAAGCCTTTAACAGGCGAAATAAAGTCAATGTGAAGGAGACGGGTACCAGGACGTTAGTCAAGGTTCAAAGAATTTCGCCCGACTTCTCTCACAGCACACACAAACCTCTAGTAACCACGGTAGTTGGAAAATATTCGAAGCGTCGTAGTCTTGGCGAAAGCAAAGACGAAAAGCGCTCGAAGAACACAAAGTGTCATAATTATGACCCTGAACGGTGGATCACTCGGCTCGTGGATCGATGAAGAGCGCAGCAAAGTGCGCTAATCCATGCGAACCGCAGAACACATGGAGCATCGAAATCTTGAACGTAAATGGCGGCCCAGCTTCACTGCTCGGGCCACATCTGACTGAGGGTCGGTTGATTGATGAACGATTATTCTGGGTGCCGAGCGGAGAGAAATTTCCATCGATGCCTTAAATTCAATCGGTCAACACTCGATGGGAGGCATTCGTAAGAGTCTGAGCGGCACCGAAGTGTCTGGAGCCCAAACGAATCTTGCTTGCTATCAACCCTGTCGGTGTTTGTGACTGTAAATGCTAAGTACAGACTCAAGTTTGCTCATAAGGCGTTTGAGGCAGTGTATAGTCTCGGACGGTACGTGTGTGTTTGTCTGTACGCCGGGCGTGTCGGGAAACTGACCACCGCGATGAGACAGCCGTACCAAGACAAATCACTGGCGAGCCTGTGAGTAGCTCAAGTCGATACAAGTACAAAGCGCTAATTTAATCATATCTGACCTCAGTTTAGGTGAGACTACCCGCTGAACTTAAGCATATCAGTAAGCGGAGGAAAAGAAACTAACAAGGATTCCCTCAGTAGCGGCGAGCGAACAGGGAAGAGCCCAGCACCGAATCCCGCGCCCTGCAAAGAGGACGCAGGGAAATGTGGTGTTTGGGAGGGCCGTGCGCGCCTGTCCGGTATTAGCCCAAGTTCTCATGAACGAGACGAGCAACCCATAGAGGGTGTCAGGCCCGTCGATAGCTAGCCGCGACCGTGCAT

> CGHna

GTTCAAACTTGATCCTTTAGAGGAAGTAAAAGTCGTAACAAGGTTTCCGTAGGTGAACCTGCGGAAGGATCATAATCGAATGAACATGTACAGTTCTAAAGCGCCTACAAGCCTTTAGACGAGTATATATTTATAGAAGCCTTTAACAGGCGAAATAAAGTCAATGTGAAGGAGACGGGTACCAGGACGTTAGTCAAGGTTCAAAGAATTTCGCCCGACTTCTCTCACAGCACACACAAACCTCTAGTAACCACGGTAGTTGGAAAATATTCGAAGCGTCGTAGTCTTGGCGAAAGCAAAGACGAAAAGCGCTCGAAGAACACAAAGTGTCATAATTATGACCCTGAACGGTGGATCACTCGGCTCGTGGATCGATGAAGAGCGCAGCAAAGTGCGCTAATCCATGCGAACCGCAGAACACATGGAGCATCGAAATCTTGAACGTAAATGGCGGCCCAGCTTCACTGCTCGGGCCACATCTGACTGAGGGTCGGTTGATTGATGAACGATTATTCTGGGTGCCGAGCGGAGAGAAATTTCCATCGATGCCTTAAATTCAATCGGTCAACACTCGATGGGAGGCATTCGTAAGAGTCTGAGCGGCACCGAAGTGTCTGGAGCCCAAACGAATCTTGCTTGCTATCAACCCTGTCGGTGTTTGTGACTGTAAATGCTAAGTACAGACTCAAGTTTGCTCATAAGGCGTTTGAGGCAGTGTATAGTCTCGGACGGTACGTGTGTGTTTGTCTGTACGCCGGGCGTGTCGGGAAACTGACCACCGCGATGAGACAGCCGTACCAAGACAAATCACTGGCGAGCCTGTGAGTAGCTCAAGTCAATACAAGTACAAAGCGCTAATCTAATCATATCTGACCTCAGTTTAGGTGAGACTACCCGCTGAACTTAAGCATATCAGTAAGCGGAGGAAAAGAAACTAACAAGGATTCCCTCAGTAGCGGCGAGCGAACAGGGAAGAGCCCAGCACCGAATCCCGCGCCCTGCAAAGAGGACGCAGGGAAATGTGGTGTTTGGGAGGGCCGTGCGCGCCTGTCCGGTATTAGCCCAAGTTCTCATGAACGAGACGAGCAACCCATAGAGGGTGTCAGGCCCGTCGATAGCTAGCCGCGACCGTGCAT

> FXHna

GTTCAAACTTGATCCTTTAGAGGAAGTAAAAGTCGTAACAAGGTTTCCGTAGGTGAACCTGCGGAAGGATCATAATCGATGAACATGTACCGTTCTTAAGCGCCCACAAGCCCTTAGACGAGTATGTATTTCTTAAAGCCTTATACGGGCGAAATAAAGTCAATGTGAAGGAGACGGGTACCAGGACGCTACGTCAAGGTTCAAAGAAATTCGCCCGACTTCTCTCACAGCACACACAAACCTCTAGTAACCACGGTAGTTGGAAAACATTCGAAACGCCATAGTCTTTGCGAAAGCAAAGACAAAAAGCGCTCGAAGAACACAATGTGTCATAATTATGACCCTGAACGGTGGATCACTCGGCTCGTGGATCGATGAAGAGCGCAGCAAAGTGCGCTAATCCATGCGAACCGCAGAACACATGGAGCATCGAAATCTTGAACGTAAATGGCGGCCCAGCTTCACTGCTCGGGCCACATCTGACTGAGGGTCGGTTGATTGATGAACGATTATTCTGGGTTCCGAGCGGAGAGAAATTTCCATCGATGCCTTAAATTCAATCGGTCAACACTCGATGGGAGGCATTCTTAAGAGTCTGAACGGCACCGAAGTGTCTGGAGCCCAAACGACTCTTATTGCTACAAACCCTGTCGGTGTTTGTGACCGTAAAAGCTAAGTACAGACTTAAGTTTGCTCATAAGGCGTTTGAGGCAGTGTAAAGTCTCGGACGGTACGTGTGTGTTTGTCTGTACGCCGGGCGTGTCGGGAAACTGACCACCGCGATGAGACAGCCGTACCAAGACAAATCACTGACGAGCCTGTGAGTAGCTCAAGTCGATACAAGTACAGAGCGCTAATCTAATCATATCTGACCTCAGTTTAGGTGAGACTACCCGCTGAACTTAAGCATATCAGTAAGCGGAGGAAAAGAAACTAACAAGGATTCCCTTAGTAGCGGCGAGCGAACAGGGAAGAGCCCAGCACCGAATCCCGCGCCCTGTAAAGAGGACGCAGGGAAATGTGGTGTTTGGGAGGGCCGTGCGCGCCTGTCCGGTATTAGCCCAAGTTCTCATGAACGAGACGAGCAACCCATAGAGGGTGTCAGGCCCGTCGATAGCTAGCCGCGACCGTGCAT

>DPHna

GTTCAAACTTGATCCTTTAGAGGAAGTAAAAGTCGTAACAAGGTTTCCGTAGGTGAACCTGCGGAAGGATCATAATCGATGAACATGTACCGTTCTTAAGCGCCCACAAGCCCTTAGACGAGTATGTATTTCTTAAAGCCTTATACGGGCGAAATAAAGTCAATGTGAAGGAGACGGGTACCAGGACGCTACGTCAAGGTTCAAAGAAATTCGCCCGACTTCTCTCACAGCACACACAAGCCTCTAGTAACCACGGTAGTTGGAAAACATTCGAAACGCCATAGTCTTTGCGAAAGCAAAGACAAAAAGCGCTCGAAGAACACAATGTGTCATAATTATGACCCTGAACGGTGGATCACTCGGCTCGTGGATCGATGAAGAGCGCAGCAAAGTGCGCTAATCCATGCGAACCGCAGAACACATGGAGCATCGAAATCTTGAACGTAAATGGCGGCCCAGCTTCACTGCTCGGGCCACATCTGACTGAGGGTCGGTTGATTGATGAACGATTATTCTGGGTGCCGAGCGGAGAGAAATTTCCATCGATGCCTTAAATTCAATCGGTCAACACTCGATGGGAGGCATTCTTAAGAGTCTGAACGGCACCGAAGTGTCTGGAGCCCAAACGACTCTTATTGCTACAAACCCTGTCGGTGTTTGTGACCGTAAAAGCTAAGTACAGACTTAAGTTTGCTCATAAGGCGTTTGAGGCAGTGTAAAGTCTCGGACGGTACGTGTGTGTTTGTCTGTACGCCGGGCGTGTCGGGAAACTGACCACCGCGATGAGACAGCCGTACCAAGACAAATCACTGACGAGCCTGTGAGTAGCTCAAGTCGATACAAGTACAGAGCGCTAATCTAATCATATCTGACCTCAGTTTAGGTGAGACTACCCGCTGAACTTAAGCATATCAGTAAGCGGAGGAAAAGAAACTAACAAGGATTCCCTTAGTAGCGGCGAGCGAACAGGGAAGAGCCCAGCACCGAATCCCGCGCCCTGTAAAGAGGACGCAGGGAAATGTGGTGTTTGGGAGGGCCGTGCGCGCCTGTCCGGTATTAGCCCAAGTTCTCATGAACGAGACGAGCAACCCATAGAGGGTGTCAGGCCCGTCGATAGCTAGCCGCGACCGTGCAT

> CNn5

GTTCAAACTTGATCCTTTAGAGGAAGTAAAAGTCGTAACAAGGTTTCCGTAGGTGAACCTGCGGAAGGATCATAATCGATGAACATGTACCGTTCTTAAGCGCCCACAAGCCCTTAGACGAGTATGTATTTCTTAAAGCCTTATACGGGCGAAATAAAGTCAATGTGAAGGAGACGGGTACCAGGACGCTACGTCAAGGTTCAAAGAAATTCGCCCGACTTCTCTCACAGCACACACAAACCTCTAGTAACCACGGTAGTTGGAAAACATTCGAAACGCCATAGTCTTTGCGAAAGCAAAGACAAAAAGCGCTCGAAGAACACAATGTGTCATAATTATGACCCTGAACGGTGGATCACTCGGCTCGTGGATCGATGAAGAGCGCAGCAAAGTGCGCTAATCCATGCGAACCGCAGAACACATGGAGCATCGAAATCTTGAACGTAAATGGCGGCCCAGCTTCACTGCTCGGGCCACATCTGACTGAGGGTCGGTTGATTGATGAACGATTATTCTGGGTGCCGAGCGGAGAGAAATTTCCATCGATGCCTTAAATTCAATCGGTCAACACTCGATGGGAGGCATTCTTAAGAGTCTGAACGGCACCGAAGTGTCTGGAGCCCAAACGACTCTTATTGCTACAAACCCTGTCGGTGTTTGTGACCGTAAAAGCTAAGTACAGACTTAAGTTTGCTCATAAGGCGTTTGAGGCAGTGTAAAGTCTCGGACGATACGTGTGTGTTTGTCTGTACGCCGGGCGTGTCGGGAAACTGACCACCGCGATGAGACAGCCGTACCAAGACAAATCACTGACGAGCCTGTGAGTAGCTCAAGTCGATACAAGTACAGAGCGCTAATCTAATCATATCTGACCTCAGTTTAGGTGAGACTACCCGCTGAACTTAAGCATATCAGTAAGCGGAGGAAAAGAAACTAACAAGGATTCCCTTAGTAGCGGCGAGCGAACAGGGAAGAGCCCAGCACCGAATCCCGCGCCCTGTAAAGAGGACGCAGGGAAATGTGGTGTTTGGGAGGGCCGTGCGCGCCTGTCCGGTATTAGCCCAAGTTCTCATGAACGAGACGAGCAACCCATAGAGGGTGTCAGGCCCGTCGATAGCTAGCCGCGACCGTGCAT
